# The Use of Defatted *Tenebrio molitor* Larvae Meal as a Main Protein Source Is Supported in European Sea Bass (*Dicentrarchus labrax*) by Data on Growth Performance, Lipid Metabolism, and Flesh Quality

**DOI:** 10.3389/fphys.2021.659567

**Published:** 2021-04-15

**Authors:** Ana Basto, Josep Calduch-Giner, Beatriz Oliveira, Lisa Petit, Tiago Sá, Margarida R. G. Maia, Susana C. Fonseca, Elisabete Matos, Jaume Pérez-Sánchez, Luisa M. P. Valente

**Affiliations:** ^1^ICBAS, Instituto de Ciências Biomédicas Abel Salazar, Universidade do Porto, Porto, Portugal; ^2^CIIMAR/CIMAR, Centro Interdisciplinar de Investigação Marinha e Ambiental, Universidade do Porto, Matosinhos, Portugal; ^3^IATS – CSIC, Instituto de Acuicultura Torre de la Sal, Castellón de la Plana, Spain; ^4^GreenUPorto, DGAOT, Faculdade de Ciências, Universidade do Porto, Porto, Portugal; ^5^SORGAL – Sociedade de Óleos e Rações, S.A., S. João de Ovar, Portugal

**Keywords:** alternative protein sources, fatty acids profile, feedstuffs, insect meal, muscle cell proliferation and differentiation, nutrient utilization

## Abstract

**Objective:**

This study aims to determine the maximal inclusion level of defatted (*d*-) *Tenebrio molitor* larvae meal (TM) able to replace dietary fishmeal (FM) without compromising growth performance, general metabolism, and flesh quality traits in European sea bass, and to evaluate the major underlying physiological mechanisms.

**Materials and Methods:**

Fish (55 ± 2 g) were fed with diets containing increasing levels of *d*TM: 0, 40, 80 and 100% (CTRL, TM40, TM80, and TM100, respectively) to replace FM. After 10 weeks of feeding, the growth performance, nutrient and energy balance, intestinal integrity, plasma metabolites and the expression of genes related to growth and nutrient metabolism, in liver and muscle were determined. The fatty acids (FA) profile, textural properties and color were also evaluated in muscle.

**Results:**

Protein and lipids digestibility remained unaltered up to 80% *d*TM inclusion. Growth performance parameters were similar among dietary treatments. The *d*TM inclusion increased the hepatosomatic index in fish fed TM100. Muscle eicosapentaenoic acid, docosahexaenoic acid and n-3 long-chain polyunsaturated FA levels were maintained up to 80% *d*TM inclusion, but total cholesterol and non-esterified FA increased with dietary *d*TM inclusion. In liver, the expression of elongation of very long-chain FA protein 6 (*elovl6*) and FA desaturase 2 (*fads2*) did not change in fish fed TM40 and TM80, but *elovl6* decreased whilst *fads2* increased in fish fed TM100 when compared to those fed CTRL. The expression of cholesterol 7 alpha-monooxygenase (*cyp7a1*) decreased with dietary *d*TM inclusion. In muscle, the expression of myoblast determination protein-2 (*myod2*) decreased in fish fed TM80 and TM100.

**Conclusion:**

It is feasible to substitute dietary FM by *d*TM up to 80% in European sea bass without detrimental effects on nutrient digestibility, growth performance and associated genetic pathways, whilst assuring fillet nutritional value for human consumption.

## Introduction

The use of insect protein as sustainable alternative to animal and plant protein sources has been encouraged for direct human consumption and incorporation into animal feeds (Nogales-Mérida et al., 2018; Salter, 2019). Insects have several advantages when compared with the conventional protein sources, as they grow fast and reproduce easily, have low feed conversion ratio (FCR) and small need of arable land and water (Gasco et al., 2020). Additionally, insects are rich protein sources, with a well-balanced essential amino acid (EAA) profile; being also rich sources of fat and some vitamins and minerals (Koutsos et al., 2019). Insects can also be valuable sources of healthy compounds, such as chitin and antioxidant and antimicrobial peptides. Recent studies showed that insect-based diets modulate fish microbiota and improve the immune system, which may reduce the use of antibiotics in aquaculture (Bruni et al., 2018: Henry et al., 2018; Antonopoulou et al., 2019; Rimoldi et al., 2019; Stenberg et al., 2019; Li et al., 2021). In 2017, European Union (EU) authorized the use of insect meal (IM) from seven species, including yellow mealworm (*Tenebrio molitor*), in aquafeeds (European Commission, 2017). Basto et al. (2020) have recently demonstrated that defatted (*d*-) *T. molitor* larvae meal (TM) is a highly digestible protein source able to meet European sea bass (*Dicentrarchus labrax*) EAA requirements (Basto et al., 2020). The potential of TM to partially replace fishmeal (FM) in aquafeeds has been previously assessed in various marine fish species, such as European sea bass or sparids (Gasco et al., 2016; Piccolo et al., 2017; Henry et al., 2018; Antonopoulou et al., 2019; Iaconisi et al., 2019; Ido et al., 2019), whilst total replacement was only evaluated in red sea bream (*Pargus major*) (Ido et al., 2019). Results among authors are controversial, and the maximal replacement level of FM by TM are extremely variable and never exceeding 71% in diets for European sea bass (Gasco et al., 2016).

The main nutritional limitation of including insects in aquafeeds is their low content of long-chain polyunsaturated fatty acids (LC-PUFA), such as the eicosapentaenoic (EPA; 20:5n−3) and docosahexaenoic (DHA; 22:6n−3) acids. Fish muscle is the main dietary source of these two n-3 LC-PUFA for humans, which are associated with beneficial health effects (Lands, 2014). Thus, IM may compromise not only flesh quality, but also growth performance when included at high levels in diets for marine fish species. Fish oil (FO) and FM are the main dietary sources of n-3 LC-PUFA, such as EPA and DHA. Thus, high FM replacement levels, or the concomitant replacement of FM and FO, have to be carefully addressed to not compromise the recommended dietary levels of n-3 LC-PUFA for marine fish species (approximately 0.7% on dry matter (DM) basis) (Skalli and Robin, 2004). In blackspot sea bream (*Pagellus bogaraveo*), full-fat TM did not affect fillet EPA or DHA content, when included at 21% of feed, but decreased the EPA content when included at 40% (Iaconisi et al., 2017). This suggest that dietary IM may induce changes in lipid metabolism, depending on its dietary inclusion level. Besides, IM also has a limited content of phosphorus (P), which may affect not only lipid metabolism, but also fish growth performance (Sugiura et al., 2004; Prabhu et al., 2014).

The present study aimed to explore the impact of partial and total replacement of FM by *d*TM in a comprehensive approach focusing not only on growth performance and nutrient utilization, but also on the underlying mechanisms involved in nutrient metabolism, namely lipid metabolism, in European sea bass. This is one of the most important fish species in Mediterranean aquaculture, where production surpassed 194,000 tons in 2018 (Eurostat, 2020). Muscle quality traits and fillet nutritional value for human consumption were also evaluated.

## Materials and Methods

### Diets

Extruded practical diets were formulated and produced by SPAROS Lda. (Portugal) to meet all the known nutritional requirements of European sea bass (NRC, 2011). All diets contained FO (12–13% inclusion level) as the main dietary lipid source. In the control diet (CTRL), FM was added at 45% and was progressively replaced in experimental diets (40%, TM40; 80%, TM80; 100%, TM100) by *d*TM from Entomo Farm (France). The inclusion level of *d*TM increased up to 60% in TM100, and was due to the concomitant replacement of FM and plant proteins, resulting in increased protein and lipid content (Table 1). All experimental diets were properly supplemented with DL-methionine and mono-calcium phosphate. For determination of the apparent digestibility coefficients (ADC), 1% chromium oxide (Cr_2_O_3_) was added, as an inert marker, to each experimental diet. Details of dietary fatty acid (FA) and amino acid (AA) profile are shown in Table 2 and Supplementary Table 1, respectively.

**TABLE 1:** Ingredients and chemical composition of the experimental diets.

	CTRL	TM40	TM80	TM100
**Ingredients, g/kg**				
Fishmeal^1^	450	270	90	–
Defatted *Tenebrio molitor* larvae meal^2^	–	180	360	600
Soy protein concentrate^3^	80	80	80	–
Soybean meal^4^	100	100	100	–
Rapeseed meal^5^	50	50	50	–
Wheat meal^6^	170	168	155	240
Sardine oil^7^	130	130	130	121
Vitamin and mineral premix^8^	10	10	10	10
Binder^9^	10	10	10	10
Mono-calcium phosphate	–	–	11	13
DL-Methionine^10^	–	2	4	6
**Chemical composition, g/100 g DM**				
Dry matter	93.5	94.3	93.2	94.2
Protein	47.8	47.7	47.0	50.6
Lipids	18.7	19.2	20.4	22.1
Energy, kJ/g DM	22.0	22.6	23.4	24.5
Phosphorus	1.4	1.1	1.0	1.0
Ash	11.7	9.0	7.4	6.5

*CTRL, control diet; DM, dry matter; TM40, TM80 and TM100, diet with 40, 80 and 100% of fishmeal replacement by defatted *Tenebrio molitor* larvae meal, respectively.*

*^1^Peruvian fishmeal super prime: 71.0% protein, 11.0% lipids, Exalmar S.A.A, Peru.*

*^2^Defatted *Tenebrio molitor* larvae meal: 65% protein, 12% lipids, 4.8% chitin, Entomo, France.^3^Soy protein concentrate: 65% protein, 0.7% lipids, ADM Animal Nutrition^TM^, The Netherlands.*

*^4^Soybean meal 48: 47.7% protein, 2.2% lipids, Cargill, Spain.*

*^5^Rapeseed meal: 36% protein, 2.7% lipids, Premix Lda., Portugal.*

*^6^Wheat meal: 10.2% protein, 1.2% lipids, Casa Lanchinha Lda., Portugal.*

*^7^Sardine oil: Sopropêche, France.*

*^8^Vitamin and mineral premix: WISIUM, ADM Portugal S.A., Portugal.*

*^9^Kielseguhr (natural zeolite): LIGRANA GmbH, Germany.*

*^10^DL-Metionine 99%: Evonik Degussa GmbH, Germany.*

**TABLE 2:** Fatty acid profile of the experimental diets.

	CTRL	TM40	TM80	TM100
**Fatty acids, g/100 g DM**				
14:00	1.1	1.1	1.1	1.2
16:00	3.1	3.3	3.4	4.2
18:00	0.7	0.7	0.7	0.9
Σ SFA^1^	5.3	5.5	5.6	6.8
16:1n-7	1.2	1.1	1.1	1.1
18:1n-9	1.6	2.2	2.8	4.7
18:1n-7	0.5	0.4	0.4	0.5
20:1n-9	0.4	0.3	0.2	0.2
22:1n-11	0.06	0.04	0.03	0.02
Σ MUFA^2^	4.4	4.7	4.8	6.8
18:2n-6	0.5	1.2	1.9	3.6
18:3n-3	0.2	0.2	0.2	0.3
18:4n-3	0.3	0.3	0.3	0.3
20:4n-6	0.1	0.1	0.1	0.1
20:5n-3, EPA	2.0	1.8	1.8	1.8
22:5n-3	0.1	0.1	0.1	0.1
22:6n-3, DHA	1.4	1.3	1.1	1.1
EPA + DHA	3.4	3.1	2.9	2.9
Σ PUFA^3^	5.4	5.6	6.2	7.9
Σ n-3 LC-PUFA^4^	4.3	3.9	3.7	3.8
Σ n-6 LC-PUFA^5^	0.7	1.4	2.1	3.8
Σ n-3/Σ n-6	5.9	2.8	1.8	1.0

*DHA, docosahexaenoic acid; DM, dry matter; EPA, eicosapentaenoic acid: LC-PUFA, long-chain PUFA; MUFA, monounsaturated fatty acids; PUFA, polyunsaturated fatty acids; SFA, saturated fatty acids.*

*^1^Includes 12:0, 13:0, 15:0, 17:0, 20:0, 20:0, 22:0, 24:0.*

*^2^Includes 14:1n-5, 16:1, 17:1n-7, 20:1n-7, 20:1n-11, 22:1n-9, 24:1n-9.*

*^3^Includes 16:2n-4, 16:3n-4, 16:4n-1, 18:2n-4, 18:3n-4, 18:3n-6, 18:4n-1, 20:2n-6, 20:3n-3, 20:4n-3, 20:3n-6, 20:3n-3, 22:2n-6, 21:5n-3 22:4n-6.*

*^4^Includes 20:3n-3, 20:4n-3, 21:5n-3.*

*^5^Includes 18:3n-6, 20:3n-6, 22:4n-6.*

### Fish Husbandry

Juvenile fish of Atlantic origin were obtained from Acuinuga S. L. (Spain). Fish, for both the digestibility and the growth trials, were held in quarantine (2000 L tanks) for 2 weeks and hand-fed with a commercial diet (AQUASOJA, Portugal – 50% crude protein and 20% crude fat on DM basis). During quarantine period, fish were held in a recirculation aquaculture system (RAS) at 24 ± 1°C, 35 ± 0.5 ‰ and 6 L/min ^–1^. Total ammonium (NH_4_^+^), nitrite (NO_2_^–^), nitrate (NO_3_^–^) and pH levels were maintained within the recommended ranges for marine species (NH_4_^+^ ≤ 0.05 mg L^–1^; NO_2_^–^ ≤ 0.5 mg L^–1^; 5 ≤ mg L^–1^; 7.5 ≤ pH ≥ 8.5). Dissolved oxygen level was kept above 90% saturation and an artificial photoperiod of 12 h’ light/12 h’ dark cycle was fixed.

### Digestibility Trial

Fish (58 ± 1 g) from the initial stock were randomly distributed in 8 tanks of 50 L (18 fish per tank) equipped with feces sedimentation columns (Cho and Slinger, 1979). Fish were fed the experimental diets (with 1% chromium oxide (Cr_2_O_3_) until apparent satiation, once a day, during 5 days for adaptation to the diets, before the feces collection began. Approximately 30 min after feeding, every tank was carefully cleaned to assure that no remains of uneaten feed were left in the bottom of the tank or in the sedimentation column. Feces were collected from the sedimentation column every morning, before feeding, and then centrifuged at 3000 *g*, to eliminate water excess, and kept at −20°C until chemical analysis. Daily collection of the feces was performed for 10 consecutive days. In order to test each diet in quadruplicate and since the RAS was only constituted by 12 tanks equipped with feces sedimentation columns, this procedure was repeated over two consecutive rounds. In each round, each diet was tested in duplicate. In the second round, diets were allocated to different tanks and fed to new groups of 18 fish per tank from the same initial stock and with the same size range (58 ± 1 g) used in the first round.

### Growth Trial

Twelve homogeneous groups of 25 fish (55 ± 2 g) from the initial stock were randomly distributed in 160 L tanks. Each diet was distributed to triplicate groups of fish, by automatic feeders until visual satiation, three times daily, for 10 weeks. The amount of feed supplied to each tank was adjusted daily, and when some uneaten feed remained in the bottom of the tank, the total amount of feed distributed each day was reduced by 5%, until no feed losses were recorded. When no feed losses were observed, the amount of feed was maintained for 2 days, and then augmented by 5%.

### Fish Sampling

After overnight fasting, ten fish from the initial stock, and five fish from each tank at the end of growth trial were sampled, sacrificed by anesthetic overdose (2-phenoxyethanol, 500 μL/L), and kept at −20°C until whole-body composition analysis. Also, at the end of the growth trial, five additional fish per tank were slightly anesthetized with 2-Phenoxyethanol (200 μL/L) for blood and tissue sampling. Blood was collected from the caudal vein with heparinized syringes, centrifuged at 10,000 *g* for 5 min at 4°C, and the collected plasma was stored at −80°C until metabolite analysis. Prior to sampling the remaining tissues, fish were sacrificed by a sharp blow on the head. Liver and white skeletal muscle (dorsal right side) portions (100 mg) were then collected, frozen in liquid nitrogen and stored at −80°C until RNA extraction. Sections of anterior intestine after the pyloric caeca (5 mm thick) were excised, washed, fixed in phosphate-buffered formalin 4% (pH 7) for 24 h, and posteriorly preserved in ethanol 70% until histological analysis. A cross-sectional slab with skin (5 mm thick) was taken just before the dorsal fin, and photographed (with a scale reference) for determination of the cross-sectional area (CSA). A portion from the left part of the fillet (1 × 1 cm) was frozen in isopentane, cooled by dry ice, and stored at −80°C for later histological analysis. Two other muscle samples, from the left part of the fillet, were taken, frozen in liquid nitrogen and stored at −80°C for DM, total lipid content and FA profile analyses. Additionally, the right dorsal fillet was collected with skin for instrumental color and texture analysis.

### Proximate Composition Analysis

Freeze-dried fish, diets and feces were ground and homogenized prior to proximate composition analysis. Ash, DM, crude protein (N × 6.25), lipids, P and energy were determined according to AOAC methods (AOAC, 2006) as described by Basto et al. (2020). The Cr_2_O_3_ content in diets and feces was determined according to Bolin et al. (1952).

### Total Lipids and Fatty Acids Analyses

Muscle total lipids were extracted and quantified gravimetrically according to Folch et al. (1957), using dichloromethane instead of chloroform. The muscle FA methyl esters (FAME) were obtained through transesterification of lipid extracts, whilst in diets FAME were obtained by direct transesterification. The identification and quantification of FAME was performed as described by Campos et al. (2017), using the tricosaenoic acid (23:0) as internal standard.

### Histological Analysis

**FIGURE 1:**
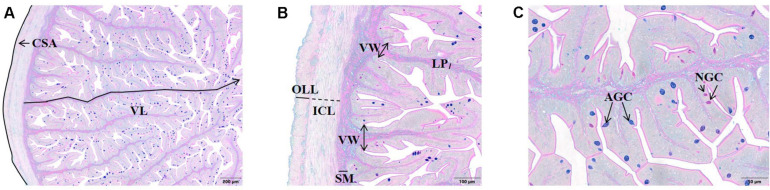
Histological sections (Alcian blue/PAS staining, pH = 2.5) of the anterior intestine of European sea bass. **(A)** CSA, cross-sectional area, VL, *villus* length (40×). **(B)** ICL, inner circular layer, LP, lamina propria, OLL, outer longitudinal layer, SM, submucosa VL, *villus* width (100×). **(C)** AGC, acid goblet cell, NGC, neutral goblet cell (200×).

Samples of anterior intestine were embedded in paraffin and processed according to standard histological procedures. Intestinal cross-sections (3 μm) were stained with specific Alcian Blue/PAS staining (pH 2.5) and examined under a light microscope (Olympus BX51, GmbH, Germany) coupled with a camera (Olympus DP50, GmbH, Germany). An imaging software (Olympus cellSens Dimension Desktop) was used to determine the following parameters: intestine CSA (mm^2^); width of *muscularis* externa (μm), outer longitudinal and inner circular layers (OLL and ICL, respectively); submucosa and lamina propria width (μm); *villus* length and width (μm); number of neutral and acid goblet cells (NGC and AGC, respectively) per *villus.* The OLL, ICL, and submucosa width were measured in eight points of each transverse section analyzed, and the mean was considered. The lamina propria width (measured in the middle of the *villus*), and *villus* length (measured from the top to the bottom following their natural curves) and width (measured at the base) were measured in the eight highest *villus.* Goblet cells were counted on these selected highest *villi.* All measurements are exemplified in Figure 1.

Frozen cross-sections of muscle (12 μm) were stained with hematoxylin-eosin and examined using the previously mentioned imaging software. The following parameters were calculated: muscle CSA (mm^2^); total number of fibers per CSA; fiber number per unit of area (density; n°/mm^2^); and frequency of fibers classes according to their diameter (≤20 μm and >140 μm) as reported in Lopes et al. (2017).

### Instrumental Texture and Color Analysis

Muscle color measurements were done with a CR-400 chromameter (Konica Minolta Inc., Japan) with an aperture of 8 mm, with respect to CIE standard illuminant D65. The chromameter was applied onto each sample and three replicates of lightness, redness and yellowness (L^∗^, a^∗^, and b^∗^, respectively) values were recorded. From a^∗^ and b^∗^ values the hue angle [H° = tan^–1^ (b^∗^/a^∗^)] and the chroma [C^∗^ = (a^*2^ + b^*2^)^1/2^] were calculated (Choubert et al., 1997). Muscle texture was analyzed using a TA.XT.plus Texture Analyser with a 5 kg load cell and a 2.0 mm diameter probe (Stable Micro systems Inc., United Kingdom). Texture profile parameters [hardness (N), adhesiveness (J), springiness (-), cohesiveness (-), chewiness (J), and resilience (-)] were obtained by double penetration (probe speed of 1 mm/s; probe penetration depth of 4 mm; wait time between penetrations of 5 s) on the thickest part of each raw fillet according to Batista et al. (2020).

### Plasma Biochemistry

Glucose, total protein, triglycerides, total cholesterol and non-esterified FA (NEFA) levels were determined enzymatically using commercial kits (1001190, 1001291, 1001313, and 1001090, Spinreact, Spain; 434-91795 NEFA-HR (2) R1 and 436-91995 NEFA-HR (2) R2, Wako Chemicals, Germany, respectively), adapting manufacturer’s instructions to microplate format.

### Gene Expression

Total RNA from liver and skeletal muscle was extracted using the MagMAX-96 total RNA isolation kit (Life Technologies, United States) after tissue homogenization in TRI reagent following manufacturers’ instructions. The RNA quantity and purity were determined by Nanodrop (Thermo Fisher Scientific, United States) with absorbance ratios (A260/A280) of 1.9–2.1. Reverse transcription (RT) of 500 ng of total RNA was performed with random decamers using the High-Capacity cDNA Archive Kit (Applied Biosystems, United States) following manufacturer’s instructions. The RT reactions were incubated for 10 min at 25°C and 2 h at 37°C. Negative control reactions were run without reverse transcriptase. The synthetized cDNA was used for PCR quantification with a SYBR Green Master Mix (Bio-Rad, Hercules, CA, United States), and specific primers at a final concentration of 0.9 μM (Supplementary Table 2). Two customized PCR-array layouts were designed for the simultaneous gene expression profiling of 49 genes covering a number of markers of growth-hormone/insulin-growth-factors (GH/IGF) system (10), lipid metabolism (12), energy metabolism (11), muscle cell proliferation and differentiation (12), and protein turnover (4) (Table 3). The program used for qPCR reactions included an initial denaturation step at 95°C for 3 min, followed by 40 cycles of denaturation for 15 s at 95°C and annealing/extension for 60 s at 60°C. All the pipetting operations were made by means of an EpMotion 5070 Liquid Handling Robot (Eppendorf, Germany) to improve data reproducibility. The efficiency of qPCRs was checked, and the specificity of reactions was verified by analysis of melting and linearity of serial dilutions of RT reactions. Fluorescence data acquired during the extension phase were normalized by the delta-delta CT method (Livak and Schmittgen, 2001), using β*-actin* as housekeeping gene due to its stability among different experimental conditions (average CT among experimental groups varied less than 0.2).

**TABLE 3:** PCR-array layout for hepatic and white skeletal muscle gene expression profiling.

Function	Gene	Symbol	Accession number^∗^
PERFORMANCE	^1,2^Growth hormone receptor-type I	*ghr-i*	AF438177
GH/IGF system	^1,2^Growth hormone receptor-type II	*ghr-ii*	AY642116
	^1,2^Insulin-like growth factor I	*igf-i*	AY800248
	^1,2^Insulin-like growth factor II	*igf-ii*	AY839105
	^1^Insulin-like binding-protein 1b	*igfbp1a*	(LG10:13787250-13788417)
	^1^Insulin-like binding-protein 2b	*igfbp2b*	EU526670
	^2^Insulin-like binding-protein 3a	*igfbp3a*	(LG4:1920612-1938180)
	^1^Insulin-like binding-protein 4	*igfbp4*	MN045298
	^2^Insulin-like binding-protein 5b	*igfpb5b*	(LG15:3836279-3847001)
	^2^Insulin-like binding-protein 6b	*igfbp6b*	(LG22-25:348158-350835)
LIPID METABOLISM	^1^Elongation of very long chain fatty acids 1	*elovl1*	KF857295
FA elongases,	^1^Elongation of very long chain fatty acids 4	*elovl4*	KF857296
FA desaturases,	^1^Elongation of very long chain fatty acids 5	*elovl5*	FR717358
Lipases,	^1^Elongation of very long chain fatty acids 6	*elovl6*	KF857297
Transcription factors	^1,2^Stearoyl-CoA desaturase 1b	*scd1b*	FN868643
	^1^Fatty acid desaturase 2	*fads2*	EU647692
	^1^Lipoprotein lipase	*lpl*	AM411614
	^1^Hepatic lipase	*hl*	KF857289
	^1^Adipose triglyceride lipase	*atgl*	KF857294
	^1^Hormone sensitive lipase	*hsl*	KF857293
	^1^Peroxisome proliferator-activated receptor α	*ppar*α	AY590300
	^1^Peroxisome proliferator-activated receptor γ	*ppar*γ	AY590303
ENERGY	^1,2^Carnitine palmitoyltransferase 1a	*cpt1a*	KF857302
METABOLISM-	^1,2^Citrate synthase	*cs*	KF857304
OXPHOS,	^1^NADH dehydrogenase subunit 5	*nd5*	KF857307
Cholesterol metabolism,	^1^Succinate dehydrogenase cytochrome b560 subunit	*sdhc*	KF857305
Energy sensing,	^1^Cytochrome b	*cyb*	EF427553
Respiration uncoupling	^1^Cytochrome c oxidase subunit I	*coxi*	KF857308
	^1^Cholesterol 7-alpha-monooxygenase	*cyp7a1*	KF857306
	^1,2^Sirtuin 1	*sirt1*	MH138004
	^1,2^Sirtuin 2	*sirt2*	MK983171
	^1^Mitochondrial respiratory uncoupling protein 1	*ucp1*	MH138003
	^2^Mitochondrial respiratory uncoupling protein 3	*ucp3*	(LG14:12134586-12136013)
MUSCLE CELL	^2^Myoblast determination protein 1	*myod1*	(LG6:934633-937237)
PROLIFERATION	^2^Myoblast determination protein 2	*myod2*	(LG5:26406310-26408511)
& DIFFERENTIATION	^2^Myogenic regulatory factor 4	*mrf4*	(LGx:14305213-14306264)
	^2^Myogenic factor 5	*myf5*	(LGx:14298644-14300040)
	^2^Myogenin	*myog*	(LG1A:13290583-13292182)
	^2^Myostatin	*mstn*	AY839106
	^2^Follistatin	*fst*	MK983166
	^2^Fibroblast growth factor 4	*fgf4*	(LG5:29962695-29967359)
	^2^Fibroblast growth factor 6	*fgf6*	AY831723
	^2^Muscle RING-finger protein 1	*murf1*	(UN:85299200-85300236)
	^2^Muscle atrophy F-box	*mafbx/atrogin-1*	MK983167
	^2^Myomaker	*mymk*	(LG20:21064893-21067975)
PROTEIN	^2^Calpain 1	*capn1*	FJ821591
TURNOVER	^2^Calpain 2	*capn2*	MK983168
	^2^Calpain 3	*capn3*	MK983169
	^2^Calpastatin	*cpst*	MK983170

*^∗^Obtained from GenBank database or the European sea bass genome project (http://seabass.mpipz.mpg.de). Accession number of sea bass genome is shown in parentheses.*

*^1^liver PCR-array.*

*^2^white skeletal muscle PCR-array.*

### Calculations

The ADC of the experimental diets were calculated according to Maynard et al. (1979): dry matter ADC (%) = 100 × [1 - (dietary Cr_2_O_3_ level/feces Cr_2_O_3_ level)] and nutrients or energy ADC (%) = 100 × [1 - (dietary Cr_2_O_3_ level/feces Cr_2_O_3_ level) × (feces nutrient or energy level/dietary nutrient or energy)]; Average body weight (ABW) = (final body weight + initial body weight)/2; Digestible nitrogen (N), lipids (L), P or energy (E) intake = (dry feed consumption × N, L, P (%) or E (kJ/g) in the diet × ADC N, L, P, or E)/ABW/days; N, L, P, or E gain = (final carcass N, L, P or E content - initial carcass N, L, P, or E content)/ABW/days; N, L, P, or E retention efficiency (NRE, LRE, PRE, or ERE) = (N, L, P, or E gain/digestible N, L, P or E intake) × 100; Fecal N, L, P, or E losses = crude N, L, P, or E intake × [1 - (ADC N, L, P, or E/100)]. Metabolic N, L, or P losses = digestible N, L, or P intake−N, L or P gain; Branchial and urinary E losses = non-fecal N losses × 25 kJ/N; Total N, L, P, or L losses = crude N, L, or P intake−N, L, or P gain; Metabolizable energy (ME) = digestible E intake - branchial and urinary E losses; Total heat loss = E intake – E gain; Total heat production = ME - energy gain. Condition factor (K) = [final body weight/(final body length)^3^] × 100; Daily growth index (DGI) = 100 × [(final body weight)^1/3^ - (initial body weight)^1/3^]/days; Voluntary feed intake (VFI) = 100 × dry feed intake/average body weight/day; Feed conversion ratio (FCR) = dry feed intake/weight gain; Protein efficiency ratio (PER) = weight gain/crude protein intake; Hepatosomatic index (HSI) = 100 × liver weight/body weight; Viscerosomatic index (VSI) = 100 × weight of viscera/body weight.

### Statistical Analysis

Data were tested for normality and homogeneity of variances by Kolmogorov-Smirnov and Levene’s tests, respectively, and log-transformed whenever required before submitted to a one-way ANOVA using IBM SPSS Statistics 26.0 (IBM corporation, United States). When one-way ANOVA showed significance (*p* < 0.05), individual means were compared using Student-Newman-Keuls post-test. Fold-changes of genes were submitted to a student’s t-test with significance set at *p* < 0.05. A bivariate spearmen’s rank correlation coefficient (*rs*) test was applied to all variables. Significant correlations were considered at the bilateral level of 0.05.

## Results

### Digestibility and Nutrient Balance

**FIGURE 2:**
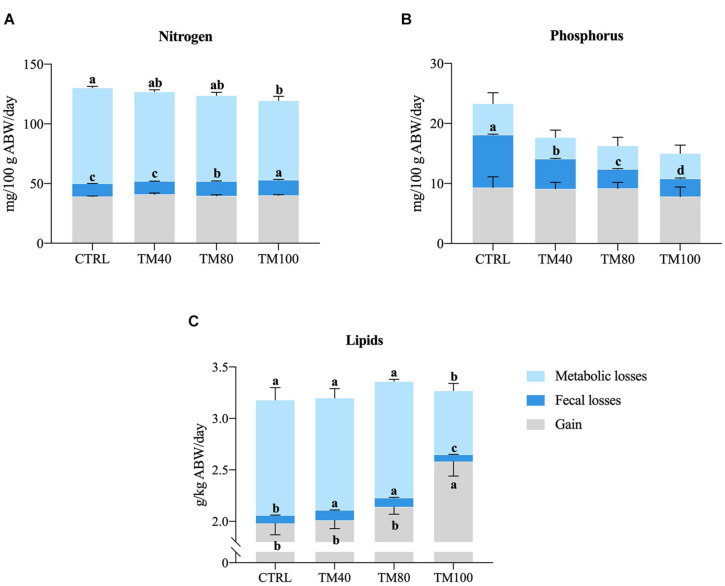
Daily nitrogen **(A)**, phosphorous **(B),** and lipids **(C)** balance of European sea bass fed experimental diets. Bars represent means ± SD; *n* = 3 (pools of 5 fish/replicate). Labeled means without a common superscript letter differ significantly, *p* < 0.05.

Overall, all diets were well digested (DM ADC > 76%; protein ADC ≥ 89%; lipids ADC ≥ 97%; energy ADC > 86%; P ADC > 62%), but differences on nutrient digestibility were found among groups in the digestibility trial (Table 4). Fish fed TM100 had the lowest protein apparent digestibility, and the highest lipid and P digestibility. As a result, N intake and metabolic N losses decreased with total FM replacement, and the opposite pattern was found for N retention efficiency. Fecal N losses increased both in fish fed TM100 and in those fed TM80. Digestible P intake decreased with FM replacement, being significantly lower in fish fed TM80 and TM100 compared to fish fed CTRL. Fecal P losses decreased significantly with the concomitant increase of dietary *d*TM. Total P losses of fish fed *d*TM decreased significantly compared to fish fed CTRL, regardless of the dietary *d*TM level. Conversely, in comparison to fish fed CTRL and other experimental groups, both lipid and energy gain and retention efficiency were consistently higher in fish fed TM100, whilst lower branchial and urinary E losses, total E losses and total heat production were observed. Fecal E losses increased significantly in fish fed TM40 and TM80. The net balance (total gain, fecal and metabolic losses) of N, P, and L is summarized in Figure 2.

**TABLE 4:** Apparent digestibility coefficients and nutrient balances of European sea bass fed experimental diets.

	CTRL	TM40	TM80	TM100	*p-*value
**ADC,** %					
Dry matter	79.0 ± 0.4^a^	76.4 ± 0.5^b^	78.3 ± 0.3^a^	78.9 ± 0.3^a^	< 0.01
Protein	91.7 ± 0.1^a^	91.3 ± 0.4^a^	90.1 ± 0.3^ab^	89.1 ± 0.9^b^	< 0.01
Lipids	97.4 ± 0.2^b^	97.0 ± 0.4^b^	97.3 ± 0.2^b^	98.0 ± 0.1^a^	0.02
Energy	87.5 ± 0.6^a^	85.7 ± 0.4^b^	86.6 ± 0.3^ab^	87.9 ± 0.4^a^	< 0.01
Phosphorus	62.4 ± 1.0^c^	71.8 ± 0.6^b^	78.6 ± 1.0^a^	80.1 ± 0.5^a^	< 0.01
**Nitrogen balance, mg/100 g ABW/day**					
Digestible N intake	119.4 ± 1.4^a^	115.9 ± 1.5^a^	111.5 ± 3.1^ab^	106.5 ± 2.7^b^	0.02
N gain	39.1 ± 0.4	40.9 ± 1.2	39.7 ± 0.8	40.0 ± 0.7	0.49
N retention efficiency,% DN	32.8 ± 0.2^b^	35.3 ± 1.0^ab^	35.6 ± 0.6^ab^	37.6 ± 1.7^a^	0.04
Fecal N losses	10.9 ± 0.1^c^	11.1 ± 0.1^c^	12.2 ± 0.4^b^	13.1 ± 0.3^a^	< 0.01
Metabolic N losses	80.3 ± 1.1^a^	74.9 ± 1.7^ab^	71.9 ± 2.6^ab^	66.5 ± 3.4^b^	0.02
Total N losses	91.2 ± 1.2	86.0 ± 1.8	84.1 ± 2.9	79.6 ± 3.7	0.08
Phosphorus balance, mg/100 g ABW/day					
Digestible P intake	14.5 ± 0.2^a^	12.7 ± 0.2^bc^	13.2 ± 0.4^b^	12.0 ± 0.3^c^	< 0.01
P gain	9.3 ± 1.8	9.1 ± 1.1	9.2 ± 1.0	7.8 ± 1.6	0.86
Phosphorus retention efficiency,% DP	64.2 ± 12.1	71.6 ± 9.0	70.6 ± 9.6	64.5 ± 12.6	0.94
Fecal P losses	8.8 ± 0.1^a^	5.0 ± 0.1^b^	3.2 ± 0.1^c^	3.0 ± 0.1^d^	< 0.01
Metabolic P losses	5.2 ± 1.8	3.6 ± 1.2	3.9 ± 1.4	4.2 ± 1.5	0.89
Total P losses	13.9 ± 1.7^a^	8.6 ± 1.2^b^	7.5 ± 1.5^b^	7.2 ± 1.4^b^	0.04
**Lipid balance, g/kg ABW/day**					
Digestible L intake	3.1 ± 0.04	3.1 ± 0.04	3.3 ± 0.1	3.2 ± 0.1	0.29
L gain	2.0 ± 0.1^b^	2.0 ± 0.1^b^	2.1 ± 0.1^b^	2.6 ± 0.1^a^	0.01
Lipids retention efficiency,% DL	63.8 ± 3.8^b^	65.0 ± 2.8^b^	65.5 ± 0.3^b^	80.6 ± 2.7^a^	< 0.01
Fecal L losses	0.08 ± 0.001^b^	0.10 ± 0.001^a^	0.09 ± 0.003^a^	0.07 ± 0.002^c^	< 0.01
Metabolic L losses	1.1 ± 0.1^a^	1.1 ± 0.1^a^	1.1 ± 0.02^a^	0.6 ± 0.1^b^	< 0.01
Total L losses	1.2 ± 0.1^a^	1.2 ± 0.1^a^	1.2 ± 0.02^a^	0.7 ± 0.1^b^	< 0.01
**Energy balance, kJ/kg ABW/day**					
Digestible E intake	327.5 ± 3.8	322.0 ± 4.0	334.6 ± 9.5	318.2 ± 8.1	0.41
E gain	123.4 ± 2.3^b^	127.3 ± 5.2^b^	128.6 ± 3.6^b^	148.6 ± 6.7^a^	0.02
Energy retention efficiency,% DE	37.7 ± 1.2^b^	39.5 ± 1.8^b^	38.5 ± 0.8^b^	46.7 ± 1.1^a^	< 0.01
Metabolizable Energy	307.5 ± 3.6	303.3 ± 3.8	316.7 ± 8.8	301.6 ± 3.7	0.39
Fecal E losses	46.9 ± 0.6^b^	53.9 ± 0.7^a^	51.5 ± 1.5^a^	43.9 ± 1.1^b^	< 0.01
Branchial + urinary E losses	20.0 ± 0.3^a^	18.7 ± 0.4^ab^	17.9 ± 0.4^ab^	16.6 ± 0.9^b^	0.02
Total E losses	250.9 ± 6.7^a^	248.7 ± 7.8^a^	257.5 ± 8.8^a^	213.4 ± 3.7^b^	0.01
Total heat production	184.0 ± 5.9^a^	176.1 ± 6.9^a^	188.1 ± 6.8^a^	153.0 ± 2.6^b^	< 0.01

*Values are means ± SEM; ADC: *n* = 4 (pools of feces from 18 fish/replicate); Nutrient balance: *n* = 3 (pools of 5 fish/replicate). Labeled means without a common superscript letter differ significantly, *p* < 0.05. ADC, apparent digestibility coefficient; DE, digestible energy; DL, digestible lipids; DN, digestible nitrogen; DP, digestible phosphorus; E, energy; L, Lipids; N, nitrogen; P, phosphorus.*

### Growth Performance and Blood Biochemistry

**FIGURE 3:**
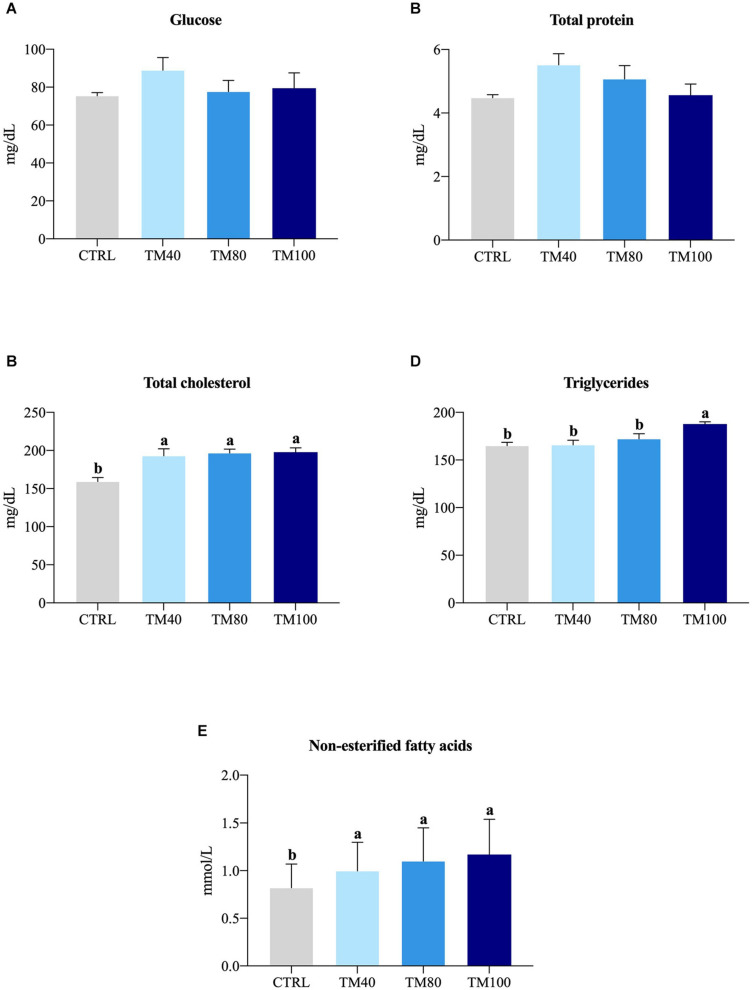
Plasma glucose **(A)**, total protein **(B)**, total cholesterol **(C)**, triglycerides **(D)** and non-esterified fatty acids **(E)** of European sea bass fed experimental diets. Bars represent means ± SEM; *n* = 15 (5 fish/replicate). Labeled means without a common superscript letter differ significantly, *p* < 0.05.

**FIGURE 4:**
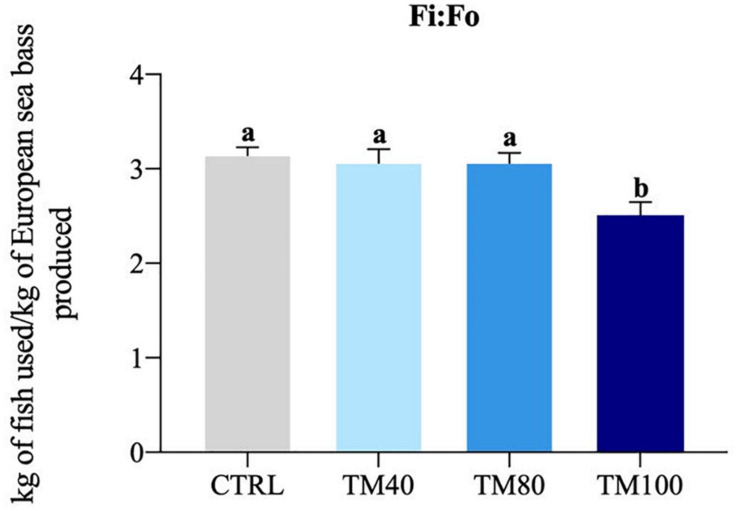
Fi:Fo ratio. Bars represent means ± SD; *n* = 3 (pools of 5 fish/replicate). Labeled means without a common superscript letter differ significantly, *p* < 0.05. Fi:Fo was calculated according to Tacon and Metian (2008), assuming yield from forage fish of 24% for FM and 4.8% for FO.

As shown in Table 5, fish almost tripled their initial weight, and no mortality was registered during the entire growth trial. All groups grew at the same rate and a significant decrease of FCR from 1.2 in fish fed CTRL to 1.0 in those fed TM100 was observed. Final K remained similar among dietary treatments. The whole-body lipids and energy content increased in fish fed TM100 with a concomitant decrease of moisture. This group also showed a statistically significant increase of HSI that was positively correlated with whole-body lipid content (0.59; *p* = 0.04) and lipid gain (0.59; *p* = 0.04). The VSI also displayed a tendency to increase in fish fed TM100 fish, being this biometric index negatively correlated with metabolic (−0.64; *p* = 0.03) and total lipid (−0.64; *p* = 0.03) losses (Supplementary Table 3). Regarding blood analysis, plasma levels of total cholesterol and NEFA increased with the replacement of FM by *d*TM, regardless of the inclusion level. Plasma triglycerides were only significantly affected by TM100, resulting in the highest values (Figure 3). Cholesterol and/or triglycerides showed positive correlations with HSI and VSI (Supplementary Table 3). Concerning the fish-in:fish-out ratio (Fi:Fo), the calculated values were reduced by 20% in fish fed TM100 in comparison to those fed CTRL or the two other experimental groups (Figure 4).

**TABLE 5:** Growth performance, somatic indexes and whole-body composition of European sea bass fed experimental diets.

	CTRL	TM40	TM80	TM100	*p-*value
**Growth performance**					
Initial body weight, g	55.6 ± 0.01	55.7 ± 0.03	55.7 ± 0.03	55.7 ± 0.02	0.31
Final body weight, g	159.4 ± 3.9	161.0 ± 3.7	159.2 ± 3.5	162.9 ± 2.2	0.86
Final K	1.3 ± 0.01	1.3 ± 0.01	1.3 ± 0.01	1.3 ± 0.02	0.09
DGI,%/day	2.4 ± 0.1	2.5 ± 0.1	2.4 ± 0.1	2.5 ± 0.04	0.87
VFI,%/day	1.7 ± 0.02^a^	1.7 ± 0.02^a^	1.6 ± 0.1^a^	1.5 ± 0.04^b^	< 0.01
FCR	1.2 ± 0.02^a^	1.1 ± 0.03^a^	1.1 ± 0.02^a^	1.0 ± 0.03^b^	0.01
PER	1.8 ± 0.03	1.9 ± 0.1	1.9 ± 0.04	2.0 ± 0.1	0.13
***Somatic indexes***					
HSI,%	1.2 ± 0.03^b^	1.1 ± 0.1^b^	1.1 ± 0.03^b^	1.5 ± 0.01^a^	0.02
VSI,%	8.1 ± 0.2	8.1 ± 0.3	7.9 ± 0.3	9.0 ± 0.3	0.06
**Whole body composition, g/100 g WW**					
Moisture	66.3 ± 0.2^a^	66.6 ± 0.5^a^	66.3 ± 0.1^a^	63.9 ± 0.9^b^	0.02
Protein	16.9 ± 0.1	17.4 ± 0.3	17.1 ± 0.03	17.0 ± 0.2	0.34
Lipids	11.4 ± 0.6^b^	11.6 ± 0.3^b^	12.2 ± 0.3^b^	14.1 ± 0.7^a^	0.02
Energy, kJ/g WW	7.7 ± 0.1^b^	7.9 ± 0.2^b^	8.0 ± 0.1^b^	8.8 ± 0.3^a^	0.02
Phosphorus	0.6 ± 0.1	0.6 ± 0.04	0.6 ± 0.1	0.6 ± 0.1	0.81
Ash	4.2 ± 0.5	3.9 ± 0.2	4.1 ± 0.3	4.1 ± 0.1	0.89

*Values are means ± SEMs; growth performance: *n* = 75 (25 fish/replicate); somatic indexes and whole-body composition: *n* = 15 (5 fish/replicate). Labeled means without a common superscript letter differ significantly, *p* < 0.05. DGI, daily growth index; FCR, feed conversion ratio; HSI, hepatosomatic index; K, condition factor; PER, protein efficiency ratio; VFI, voluntary feed intake; VSI, viscerosomatic index; WW, wet weight.*

### Muscle Total Lipids and Fatty Acids Analyses

Despite the increase of lipids in TM100 diet, the muscle total lipids content was similar among groups (Table 6). The muscle saturated (SFA) and monounsaturated FA (MUFA) profile reflected the dietary FA profile (Table 2). Up to 80% of FM replacement by *d*TM, the muscle PUFA profile also reflected the dietary FA profile. On the other hand, and despite the highest percentage of PUFA in TM100 diet, muscle PUFA levels in fish fed TM100 were comparable to those of all other treatments. The relative percentage of muscle EPA, DHA, and n-3 LC-PUFA of fish fed TM40 and TM80 was similar to those fed CTRL, despite their dietary percentage being decreased with the *d*TM inclusion. Although fish fed TM100 had the lowest relative percentage of EPA and DHA, when expressed in wet weight (g/100 g WW), both muscle EPA and EPA + DHA final contents were similar among all fish. As observed in the experimental diets, the percentage of muscle n-6 LC-PUFA increased with *d*TM inclusion and consequently the n-3/n-6 ratio decreased.

**TABLE 6:** Muscle total lipid content and fatty acid composition of the European sea bass fed experimental diets.

	CTRL	TM40	TM80	TM100	*p*-value
Total lipids, g/100 g WW	2.5 ± 0.1	2.5 ± 0.1	2.6 ± 0.3	2.8 ± 0.2	0.73
**Fatty acids, g/100 g total fatty acids**					
14:0	4.2 ± 0.1	4.1 ± 0.04	4.0 ± 0.3	3.6 ± 0.04	0.05
16:0	17.8 ± 0.03	17.6 ± 0.1	17.6 ± 0.2	17.8 ± 0.1	0.74
18:0	3.5 ± 0.06	3.7 ± 0.05	3.7 ± 0.05	3.9 ± 0.02	0.08
Σ SFA^1^	27.4 ± 0.1	27.3 ± 0.1	27.1 ± 0.4	26.9 ± 0.1	0.81
16:1n-7	6.1 ± 0.2^a^	5.6 ± 0.02^a^	5.6 ± 0.2^a^	4.5 ± 0.03^b^	0.01
18:1n-9	17.5 ± 0.5^b^	17.3 ± 0.1^b^	18.0 ± 0.5^b^	22.4 ± 0.1^a^	0.01
18:1n-7	2.9 ± 0.1^a^	2.7 ± 0.02^ab^	2.6 ± 0.1^ab^	2.2 ± 0.004^b^	0.04
20:1n-9	2.0 ± 0.2	1.9 ± 0.02	1.7 ± 0.2	1.2 ± 0.003	0.08
22:1n-11	1.3 ± 0.2	1.2 ± 0.01	1.1 ± 0.2	0.5 ± 0.004	0.07
Σ MUFA^2^	31.7 ± 0.3	30.6 ± 0.1	30.8 ± 0.3	32.2 ± 0.2	0.05
18:2n-6	4.6 ± 0.04^d^	7.1 ± 0.1^c^	9.9 ± 0.005^b^	13.5 ± 0.2^a^	< 0.01
18:3n-3	1.1 ± 0.01	1.1 ± 0.01	1.1 ± 0.02	1.1 ± 0.01	0.23
18:4n-3	1.3 ± 0.1^a^	1.2 ± 0.01^a^	1.2 ± 0.1^a^	0.9 ± 0.003^b^	0.01
20:4n-6	0.9 ± 0.02^a^	1.0 ± 0.02^a^	0.9 ± 0.01^a^	0.7 ± 0.01^b^	< 0.01
20:5n-3, EPA	10.2 ± 0.2^a^	10.0 ± 0.05^a^	9.9 ± 0.2^a^	7.9 ± 0.1^b^	< 0.01
22:5n-3	1.8 ± 0.04^a^	1.8 ± 0.02^a^	1.8 ± 0.02^a^	1.3 ± 0.2^b^	< 0.01
22:6n-3, DHA	11.6 ± 0.3^a^	12.6 ± 0.1^a^	11.7 ± 0.1^a^	9.0 ± 0.2^b^	< 0.01
EPA + DHA	21.8 ± 0.5^a^	22.6 ± 0.1^a^	21.6 ± 0.1^a^	16.9 ± 0.1^b^	< 0.01
Σ PUFA^3^	35.0 ± 0.6^b^	37.9 ± 0.1^a^	39.7 ± 0.5^a^	37.3 ± 0.2^ab^	< 0.01
Σ n-3 LC-PUFA^4^	27.1 ± 0.6^a^	27.7 ± 0.2^a^	26.7 ± 0.4^a^	21.1 ± 0.3^b^	< 0.01
Σ n-6 LC-PUFA^5^	6.4 ± 0.1^d^	9.0 ± 0.1^c^	11.7 ± 0.02^b^	15.2 ± 0.2^a^	< 0.01
Σ n-3/Σ n-6	4.2 ± 0.1^a^	3.1 ± 0.05^b^	2.3 ± 0.04^c^	1.4 ± 0.03^d^	< 0.01
**Fatty acids, g/100 g WW**					
20:5n-3, EPA	0.22 ± 0.01	0.20 ± 0.01	0.21 ± 0.02	0.20 ± 0.01	0.46
22:6n-3, DHA	0.25 ± 0.01^a^	0.24 ± 0.01^a^	0.25 ± 0.02^a^	0.18 ± 0.01^b^	< 0.01
EPA + DHA	0.47 ± 0.02	0.44 ± 0.01	0.46 ± 0.04	0.38 ± 0.01	0.38

*Values are means ± SEMs; Total lipids: *n* = 15 (5 fish/replicate); Fatty acids: *n* = 6 (2 pools/replicate). Labeled means without a common superscript letter differ significantly, *p* < 0.05. DHA, docosahexaenoic acid; EPA, eicosapentaenoic acid; LC-PUFA, long-chain PUFA; MUFA, monounsaturated fatty acids; PUFA, polyunsaturated fatty acids; SFA, saturated fatty acids; WW, wet weight.*

*^1^Includes 12:0, 13:0, 15:0, 17:0, 20:0, 20:0, 22:0, 24:0.*

*^2^Includes 14:1n-5, 16:1, 17:1n-7, 20:1n-7, 20:1n-11, 22:1n-9, 24:1n-9.*

*^3^Includes 16:2n-4, 16:3n-4, 16:4n-1, 18:2n-4, 18:3n-4, 18:3n-6, 18:4n-1, 20:2n-6, 20:3n-3, 20:4n-3, 20:3n-6, 20:3n-^3^22:2n-6, 21:5n-3; 22:4n-6.*

*^4^Includes 20:3n-3, 20:4n-3, 21:5n-3.*

*^5^Includes 18:3n-6, 20:3n-6, 22:4n-6.*

### Intestine and Muscle Histological Analysis

The morphology of anterior intestine was well-preserved in all fish, with no signs of *villus* fusion or enterocyte vacuolization. Intestine CSA varied between 11 and 15 mm^2^. The OLL and ICL varied between 32 and 41 μm, and 58 and 69 μm, respectively. The lamina propria width varied between 27 and 30 μm. The *villus* length and width ranged from 1749 to 2001 μm and 161 to 180 μm, respectively. The number of AGC and NGC per *villus* ranged from 147 to 192, and 70 to 113, respectively. The morphometric parameters analyzed in the anterior intestine did not vary significantly among dietary treatments, with the exception of submucosa thickness that increased concomitantly with dietary *d*TM inclusion level: the submucosa thickness increased from 27 μm in fish fed CTRL to 29, 30, and 33 μm in those fed TM40, TM80 and TM100, respectively (Supplementary Table 4). White muscle CSA varied between 459 and 496 mm^2^, total number of fibers ranged from 104 to 108 thousand, and fiber density from 217 to 228 (Supplementary Table 5). Fiber diameter varied between 65 and 67 μm, with 5–6% of the fibers having below 20 μm and 2–3% above 140 μm. None of the morphometric parameters analyzed in muscle had significant differences among dietary treatments.

### Muscle Instrumental Color and Texture Analysis

Texture and color parameters were similar among dietary treatments (Supplementary Table 6). Hardness ranged from 0.9 to 1 N, adhesiveness from −0.01 to −0.004 J, springiness from 1.2 to 1.6, cohesiveness from 0.4 to 0.5, chewiness from 0.5 to 0.8 J, and resilience was 0.4 irrespectively of the dietary treatment. In the muscle, L^∗^ varied from 41 to 42, a^∗^ from −0.5 to −0.2, b^∗^ from −0.7 to 0.1, H° from 183 to 250 and C° from 1 to 1.2.

### Gene Expression

**FIGURE 5:**
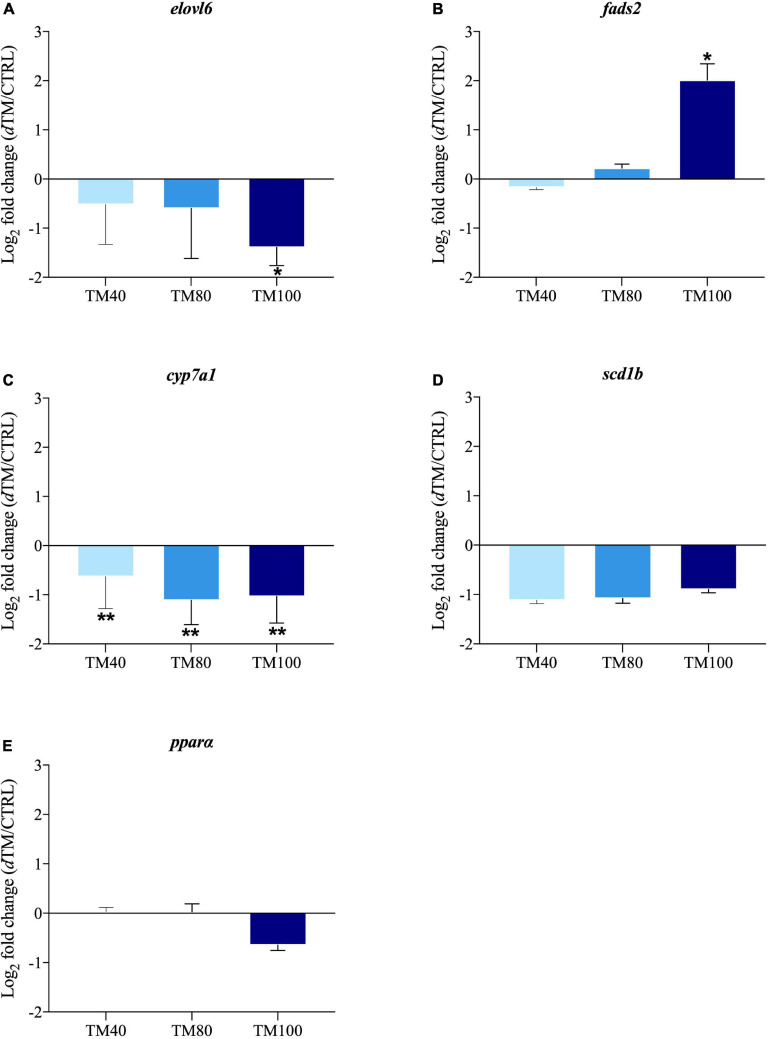
Fold-changes (*d*TM/CTRL) of differentially expressed genes in liver tissue: *elovl6* **(A)**, *fads* **(B)**, *cyp7a1* **(C)**, *scd1b* **(D)** and *ppar*α **(E)**. The asterisks indicate statistically significant differences (*p < 0.05; **p < 0.01; t-student) between European sea bass fed *d*TM and CTRL diets. Values >1 indicate up-regulated genes in *d*TM fish; values <1 indicate down-regulated genes in *d*TM fish; *n* = 9 (3 fish/replicate).

**FIGURE 6:**
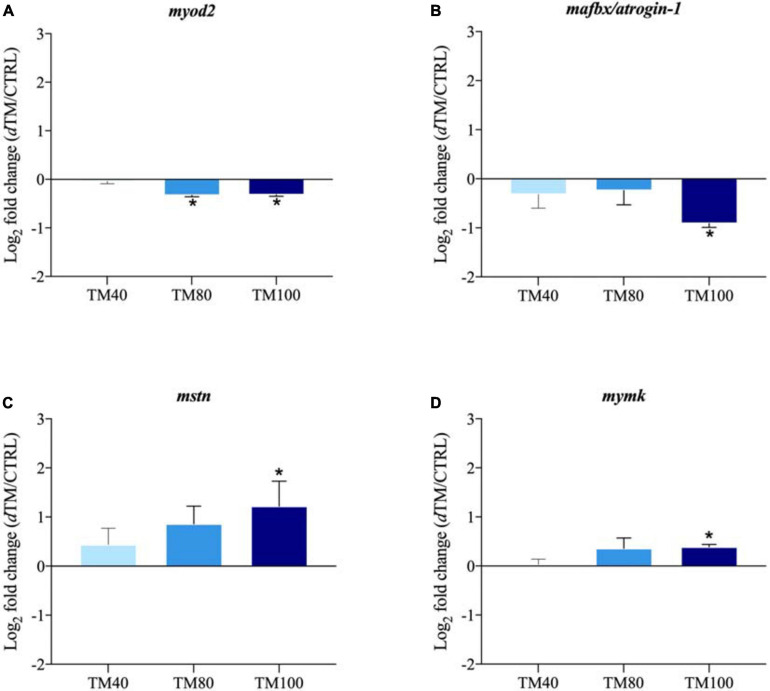
Fold-changes (*d*TM/CTRL) of differentially expressed genes in muscle tissue: *myod2* **(A)**, *mstn* **(B)**, *mafbx/atrogin* 1 **(C)**, and *mymk* **(D)**. The asterisks indicate statistically significant differences (*p < 0.05; t-student) between European sea bass fed *d*TM and CTRL diets. Values >1 indicate up-regulated genes in *d*TM fish; values <1 indicate down-regulated genes in *d*TM fish. *n* = 9 (3 fish/replicate).

The relative gene expression profile of key selected markers in liver and muscle is shown in Supplementary Tables 7, 8, with arbitrarily assigned values of 1 for hormone sensitive lipase (*hsl*) in liver of fish fed CTRL, and calpain 2 (*capn2*) in muscle of fish fed CTRL. Among all the analyzed genes, up to three markers of hepatic lipid metabolism and four markers of muscle growth were differentially expressed with the FM replacement, and the corresponding log_2_ fold-changes (*d*TM/CTRL) were graphically represented. In liver, the expression of elongation of very long-chain FA protein 6 (*elovl6)* was significantly down-regulated in fish fed TM100, but not in those fed TM40 and TM80 (Figure 5A). Conversely, FA desaturase 2 (*fads2)* was markedly up-regulated in fish fed TM100, remaining almost unaltered in those fed TM40 and TM80 (Figure 5B). A strong nutritional regulation was also found for cholesterol 7-alpha-monooxygenase (*cyp7a1*) with a significant down-regulation in all *d*TM groups (Figure 5C). A similar trend was observed for the expression of stearoyl-CoA desaturase 1b (*scd1b*), although this down-regulation was not statistically significant (Figure 5D). The expression of lipolytic peroxisome proliferator-activated receptor α (*ppar*α) also evidenced a non-significant down-regulation trend for fish fed TM100 (Figure 5E). The expression of e*lovl6*, *cyp7a1* and *ppar*α was negatively correlated with plasma cholesterol levels (−0.64 (*p* < 0.01), −0.46 (*p* < 0.01), and −0.37 (*p* = 0.02), respectively) (Supplementary Table 9). Myoblast determination protein 2 (*myod2*) was down-regulated in TM80 and TM100 (Figure 6A), whereas a significant down-regulation of muscle atrophy F-box (*mafbx/atrogin-1*) was only found in fish fed TM100 (Figure 6B). Contrarily, the expression of myostatin (*mstn*) (Figure 6C) and myoblast fusion factor (*mymk*) (Figure 6D) tended to increase with FM replacement level, but this increase was only statistically significant in TM100.

## Discussion

Since the use of IM in aquafeeds was authorized by EU in 2017, increasing efforts have been made to identify the best insect species to substitute FM, and its highest dietary inclusion level (Gasco et al., 2020). Despite the current high market prices of insect meal compared to conventional protein sources (Arru et al., 2019), the insect production is a fast-growing sector, and several companies or start-ups have been recently founded in Europe. This may lead to increased price competitiveness in the near future. The present study clearly shows that the total substitution of FM by *d*TM can be achieved in European sea bass without impairing growth performance or condition factor and even improving FCR after 10 weeks of feeding. To the best of our knowledge, the maximum replacement of FM by full-fat IM, successfully tested in European sea bass, varied between 36% and 50%, in trials of similar duration (Gasco et al., 2016; Abdel-Tawwab et al., 2020). Skalli and Robin (2004) pointed out that growth impairment at higher FM replacement levels is primarily due to nutrient deficiencies in n-3 LC-PUFA, which are fixed for marine fish close to 0.7% on DM basis (Skalli and Robin, 2004). In fact, a previous study in sea bass with 71% FM replacement by TM did not fulfil this requirement, impairing growth (Gasco et al., 2016), whilst all experimental diets tested in the present study contained 3.8–4.3% DM n-3 LC-PUFA, probably due to the high quality of FO, and did not affect growth performance. In blackspot sea bream and gilthead sea bream (*Sparus aurata*), it was possible to replace FM by full-fat TM up to 49% and 74%, respectively, without affecting growth performance or FCR, after 18–23 weeks of feeding (Iaconisi et al., 2017; Piccolo et al., 2017). In red sea bream, the total replacement of FM by *d*TM was successfully achieved without affecting FCR and even increasing growth after 4 weeks of feeding (Ido et al., 2019). Contrarily, the use of defatted black soldier fly (*Hermetia illucens)* to replace FM at levels above 18% in turbot (*Psetta maxima*) and 35% in meagre (*Argyrosomus regius*), for 7–9 weeks, did not affect FCR, but impaired growth performance (Kroeckel et al., 2012; Guerreiro et al., 2020). Thus, optimal FM substitution levels could vary among fish, insect species, rearing conditions, processing methods of IM, and duration of the experimental trials making comparisons difficult (Osimani et al., 2016; Iaconisi et al., 2019). Therefore, standardization of insect protein production is needed to assure the correct formulation of diets not only for fish, but also for other animal species.

In the present study, up to 80% of FM replacement did not alter N intake, N retention or metabolic N losses. However, this trade-off was apparently altered in fish fed TM100; these fish conserved a higher N retention compared to control by reducing metabolic N losses. Digestibility disturbances with the use of IM are attributed to the chitin, which has been negatively correlated with protein digestibility in *in vitro* digestibility studies (Marono et al., 2015). Although chitinolytic activity was reported in some fish species (Gutowska et al., 2004; Krogdahl et al., 2005; Kawashima et al., 2016), there are evidences that high dietary chitin levels have negative impacts on fish growth and nutrient utilization (Alegbeleye et al., 2012; Kroeckel et al., 2012). In the present study, this negative impact seems to have apparently been mitigated by the low chitin content (4.8% DM) in *d*TM. Moreover, the integrity of anterior intestine was well preserved in all fish, without major morphological changes, with the exception of submucosa thickening in fish fed *d*TM. Li et al. (2020) have observed a higher degree of submucosa cellularity in the anterior intestine of seawater-phase Atlantic salmon (*Salmo salar*) fed a *H. illucens* based diet, but the submucosa of mid and posterior intestine remained unaltered after 8 weeks of feeding such diets to freshwater-phase fish (Li et al., 2019). According to Sitjà-Bobadilla et al. (2005), the increase in thickness of submucosa is usually due to infiltrations of granular eosinophil cells, but this could not be confirmed in the present study. In contrast to the present results, in rainbow trout (*Oncorhynchus mykiss*), the replacement of FM by 25–50% full-fat or 100% defatted *H. illucens* reduced *villus* length in the anterior intestine after 12–14 weeks of feeding (Dumas et al., 2018; Cardinaletti et al., 2019). These controversial results may be associated to different species, feeds and processing methods used for IM production, leading to different nutritional profiles.

It was also observed that P ADC increased from 62% in fish fed CTRL to 72–80% in those fed the *d*TM diets. This higher P digestibility resulted in a pronounced reduction of total P losses, which suggests that the use of *d*TM as a FM replacer in European sea bass could help reducing P output into the environment. Likewise, Wang et al. (2017) demonstrated in Nile tilapia that FM replacement by increasing levels of *M. domestica* from 18% to 100% resulted in lower concentrations of total phosphate in the water. This metabolic feature might be part of the adaptive response of fish facing a reduced dietary P supply. Indeed, P deficiencies are a common nutritional disturbance of FM replacement by alternative proteins in aquafeeds (Sugiura et al., 2004; Prabhu et al., 2014). In the case of IM, this is a relevant issue because the mineral content of this feedstuff is markedly lower than FM or other conventionally FM substitutes, such as plant proteins. Indeed, ash content was reduced herein from 11.7% in CTRL diet to 6.5% in TM100 diet.

With total FM replacement, the increase of dietary lipids and energy content was concomitant with an improved digestibility of lipids that would favor increased whole-body lipid content by the end of the growth trial. This trend was not found in previous European sea bass studies where FM was replaced by IM from 25% to 71% (Gasco et al., 2016; Magalhães et al., 2017; Abdel-Tawwab et al., 2020). Likewise, in rainbow trout, Rema et al. (2019) did not observe any impact of *d*TM on lipid digestibility and retention. Regardless of this, our data of tissue body fat depots suggest an enhanced flux of lipids toward liver and secondly mesenteric fat when FM is totally replaced by *d*TM. This assumption is supported by data on muscle lipid content, and circulating lipid levels that were positively correlated with HSI and VSI. This is not surprising since European sea bass is a fish with low to moderate lipid deposition rates in the fillet (Ballester-Lozano et al., 2016). Although lipid content of liver has not been assessed, the strong and positive correlation between circulating triglycerides and HSI, and the moderate and positive correlation between final whole-body lipids content, lipid gain and HSI may indicate liver steatosis which is a sign of deficiencies in minerals and n-3 LC-PUFA (Ballester-Lozano et al., 2015). In contrast, enhanced deposition rate in mesenteric fat depots is highly informative of P deficiencies (Sugiura et al., 2004). In any case, both mineral and lipid metabolism seem to be highly sensitive to FM replacement by IM, which might be exacerbated at long-term through the production cycle, especially in the case of lipid-enriched diets (see below).

Liver plays a major role in fish lipid metabolism, and its gene expression profile is greatly influenced by dietary composition and feeding levels (Monroig et al., 2018). In the present study, all diets were formulated with 12–13% of FO and the n-3 LC-PUFA levels (3.8–4.3% DM) were above recommended level for marine fish species (approximately 0.7% DM) (Skalli and Robin, 2004). When revisiting the gene expression profile of gilthead sea bream fed isolipidic diets with a maximal replacement of FO by vegetable oils or semisynthetic diets formulated to be deficient in n-3 LC-PUFA, the most responsive hepatic enzymes of lipid metabolism are *scd1* and *elovl6*, and secondly *fads2* (Benedito-Palos et al., 2016). This will contribute to mitigate the signs of deficiencies in n-3 LC-PUFA, though it is very important to limit hepatic lipogenesis to avoid the lipotoxic effects of excessive fat accumulation (Perera et al., 2019). Indeed, *scd1* is the rate limiting enzyme in the synthesis of MUFA, especially oleic acid (OA, 18:1n-9) and palmitoleic acid (16:1n-7) from stearoyl-CoA and palmitoyl-CoA, respectively. Likewise, *elovl6* is responsible for the elongation of SFA and MUFA of 12, 14 and 16 carbons to form 18-carbon FA (Weiss-Hersh et al., 2020). Therefore, the up-regulation of *scd1b* and *elovl6* enhances the biosynthesis of MUFA, which in turn increases the unsaturation index of FA membrane phospholipids. This is reinforced by the recent observation of Perera et al. (2019) that *scd1a* is epigenetically regulated in gilthead sea bream by broodstock nutrition with alpha-linolenic acid (ALA, 18:3n-3) enriched diets. In the present study, no changes in ALA were found among diets, whilst both OA and linoleic acid (LA, 18:2n-6) were 3–7 times higher in TM100 than in the CTRL diet. The observed down-regulation of *elovl6* in fish fed TM100, accompanied by a global decreasing trend of *scd1b* expression with FM replacement, support a reduced *de novo* lipogenic activity. In this scenario, the simultaneous up-regulated expression of *fads2* in fish fed TM100 will serve to further desaturate PUFA, but the capacity of *de novo* synthesis of n-3 LC-PUFA is limited in marine fish and European sea bass in particular, due to the lack of *elovl2* and reduced delta 5 desaturase activity (Monroig et al., 2018). Thus, although all dietary treatments provided European sea bass recommended n-3 LC-PUFA levels, TM100 diet may compromise the muscle n-3 LC-PUFA content at long term.

Cholesterol metabolism is also affected by FM replacement, and the hypocholesterolemic effect of plant proteins as FM replacers has been observed in a wide range of fish species, including gilthead sea bream (Gómez-Requeni et al., 2004; Benedito-Palos et al., 2016), European sea bass (Messina et al., 2013), rainbow trout (Romarheim et al., 2008) and salmon (Hartviksen et al., 2014). Likewise, feeding trials conducted with semisynthetic diets highlighted that FO replacement with vegetal oils acts lowering triglycerides and cholesterol in gilthead sea bream (Ballester-Lozano et al., 2015). In the same study, diets formulated for deficiencies in phospholipids and vitamins also showed a hypocholesterolemic effect, whereas P deficiency acted as a hypercholesterolemic factor. Since this same trend was found herein with FM replacement by *d*TM, the availability of bioactive P becomes again a limiting factor for the successful replacement of FM, though signs of growth and health impairment were mostly masked at short-term. The precise mechanism remains unknown, but it would be favored by the down-regulated expression of hepatic *cyp7a1*, the rate limiting enzyme of the bile acid synthesis pathway (Jelinek et al., 1990). This assumption was supported by the negative correlation between circulating cholesterol and the expression level of hepatic *cyp7a1*, as *cyp7a1* deficiency has been reported to lead to hypercholesterolemia (Pullinger et al., 2002; Erickson et al., 2003; Qayyum et al., 2018). The ultimate physiological consequences of these findings remain elusive, but intriguingly the suppression of the hepatic *cyp7a1* expression in mice fed cholesterolemic diets mainly involves the activation of inflammatory cytokines (Henkel et al., 2011).

Growth of fish is intrinsically related to protein deposition in muscle, which is regulated by systemic and local signaling pathways involving the GH/IGF system (Mommsen, 2001). In this study, no differences in the expression of *gh* receptors or *igfs* transcripts were observed among diets, which is in line with growth performance results. Nonetheless, *myod2* plays a pivotal role in myoblast proliferation and differentiation (Valente et al., 2013), and its down-regulated expression in fish fed TM80 and TM100 should be indicative of some muscle growth derangement. Moreover, *mstn* is a negative regulator of muscle growth through inhibition of proliferation and differentiation of myogenic progenitor cells (Bonnieu et al., 2007), and its up-regulation coupled to the down-regulated expression of *myod2* highly supports muscle growth inhibition at the transcriptional level. The extent to which this negative feature is counter-regulated by compensatory growth mechanisms remains uncertain, but the expression of key transcripts of the ubiquitin proteasome pathway, with a main role in the protein turnover of skeletal muscle (Foletta et al., 2011), remained unaltered (muscle RING-finger protein-1; *murf1*) or were even down-regulated (*mafbx/atrogin-1*) with the total FM replacement. Furthermore, a recent study in zebrafish demonstrated that *mymk* plays a pivotal role in myoblast fusion and consequently in muscle growth (Shi et al., 2018), and interestingly the expression of this growth-promoting factor was consistently up-regulated with the total FM replacement. The net result at short-term would be, thereby, the preservation of muscle growth with the use of IM as a main protein source in European sea bass. This was supported by the lack of changes in skeletal muscle cellularity and instrumental texture properties. To our best knowledge, the muscle cellularity of fish fed IM diets was never assessed before, but previous studies in different farmed fish species have evidenced the preservation of textural properties with high levels of FM replacement by IM (Lock et al., 2016; Borgogno et al., 2017; Iaconisi et al., 2017; Wang et al., 2017; Secci et al., 2019; Bruni et al., 2020).

In conclusion, the results of the present study demonstrate that FM replacement by *d*TM is largely feasible in European sea bass without detrimental effects on growth performance, nutrient utilization, intestinal integrity, and flesh nutritional and textural quality. Indeed, FM replacement by *d*TM resulted in fair levels of EPA and DHA in muscle of European sea bass (0.38–0.46 g/100 g WW), which are above those recommended by EFSA (2010) for human consumption to decrease the risk of cardiovascular diseases (0.25 g of EPA plus DHA per 100 g of fish). Also, most of the possible negative effects on P and lipid metabolism are largely mitigated at this high replacement level. Nonetheless, further research is needed to fully validate such nutritional approach at farm level throughout the production cycle, giving special attention to the total energy content of the diet since lipid-energized diets can exacerbate the disturbance of lipid metabolism. It is also important to mention that the reduction of P emissions to the environment, coupled with the reduction in the Fi:Fo ratio, prove that *d*TM is an environmentally sustainable alternative to FM.

## Data Availability Statement

The raw data supporting the conclusions of this article will be made available by the authors, without undue reservation.

## Ethics Statement

The animal study was reviewed and approved by IATS-CSIC and CSIC Review Boards, European (2010/63/EU) Animal Directives, and Spanish Laws (Royal Decree RD53/2013) on the handling of experimental animals.

## Author Contributions

LV, JP-S, JC-G, and EM conceived and designed the study. AB, BO, LP, TS, MM, and SF conducted research, analyzed data, and performed statistical analysis. AB, JP-S, and LV wrote the article and had primary responsibility for final content. All authors read and approved the final manuscript.

## Conflict of Interest

EM was employed by the company SORGAL – Sociedade de Óleos e Rações, S.A., S. João de Ovar, Portugal. The remaining authors declare that the research was conducted in the absence of any commercial or financial relationships that could be construed as a potential conflict of interest.
