# Supplementary material for: The Use of Defatted Tenebrio molitor Larvae Meal as a Main Protein Source Is Supported in European Sea Bass (Dicentrarchus labrax) by Data on Growth Performance, Lipid Metabolism, and Flesh Quality
Source: Front Physiol. 2021 Apr 15;12:659567. doi: 10.3389/fphys.2021.659567 (PMC8104126; doi:10.3389/fphys.2021.659567)
Supplement: Supplementary file 1 [file Table_1.docx]

| **SUPPLEMENTARY TABLE 1 \|** Amino acid profile of the experimental diets | | | | |
| --- | --- | --- | --- | --- |
|  | CTRL | TM40 | TM80 | TM100 |
| ***Amino acids, g/100 g DM*** | | |  |  |
| Arginine | 3.8 | 3.7 | 3.8 | 3.6 |
| Histidine | 1.1 | 1.4 | 1.4 | 1.5 |
| Lysine | 3.3 | 2.8 | 2.6 | 3.2 |
| Threonine | 1.7 | 2.0 | 1.8 | 1.8 |
| Isoleucine | 2.1 | 2.3 | 2.3 | 2.4 |
| Leucine | 3.8 | 3.5 | 3.6 | 7.0 |
| Valine | 2.4 | 2.6 | 2.9 | 3.5 |
| Methionine | 1.5 | 1.4 | 1.3 | 1.6 |
| Phenylalanine | 2.5 | 2.3 | 2.2 | 2.1 |
| Cystine | 0.3 | 0.3 | 0.3 | 0.3 |
| Tyrosine | 2.4 | 2.6 | 2.9 | 3.6 |
| Aspartic acid + Asparagine | 4.1 | 4.0 | 3.9 | 4.2 |
| Glutamic acid + Glutamine | 7.8 | 6.4 | 6.5 | 6.2 |
| Alanine | 2.5 | 2.8 | 2.9 | 3.5 |
| Glycine | 3.2 | 3.3 | 3.2 | 3.3 |
| Proline | 3.3 | 3.0 | 2.9 | 3.5 |
| Serine | 1.9 | 2.0 | 1.9 | 1.7 |
| Taurine | 0.2 | 0.2 | 0.06 | 0.04 |
| DM, dry matter. | | | | |

**SUPPLEMENTARY MATERIAL**

| **SUPPLEMENTARY TABLE 2** \| Forward and reverse primers for real-time qPCR | | | | | | |
| --- | --- | --- | --- | --- | --- | --- |
| **Gene** | **Symbol** | **Accession number^1^** | **Primers** |  |  |  |
|  |  |  |  |  |  |  |
| Growth hormone receptor-type I | *ghr-i* | AF438177 | F: GGT GGA TGC TGA GGA TGC | | | |
|  |  |  | R: GGT GTC TGA GCC CTG GTT | | | |
|  |  |  |  |  |  |  |
| Growth hormone receptor-type II | *ghr-ii* | AY642116 | F: TCC AGT CCA GAG CCC TAC | | | |
|  |  |  | R: ACG ACC TCA CCT CAC TCA | | | |
|  |  |  |  |  |  |  |
| Insulin-like growth factor I | *igf-i* | AY800248 | F: TAG CCA CAC CCT CTC ACT ACT G | | | |
|  |  |  | R: CCT GTT GCC GTC GGA GTC | | | |
|  |  |  |  |  |  |  |
| Insulin-like growth factor II | *igf-ii* | AY839105 | F: AGA CAC GGA CAC CAC ACA CTT TG | | | |
|  |  |  | R: CTC TTG ACC TTC ATT CTG CTG CTC TC | | | |
|  |  |  |  |  |  |  |
| Insulin-like binding-protein 1b | *igfbp1a* | (LG10:13787250-13788417) | F: AGT GTG AAT CAT CTC TGG TTG GA | | | |
|  |  |  | R: CCC ATT CCA GGA AGA GAC ACA | | | |
|  |  |  |  |  |  |  |
| Insulin-like binding-protein 2b | *igfbp2b* | EU526670 | F: GCA CGG AGG CTG ACT TAC C | | | |
|  |  |  | R: CTT GGT CCA GAG TTG TTG TGA GAT | | | |
|  |  |  |  |  |  |  |
| Insulin-like binding-protein 3a | *igfbp3a* | (LG4:1920612-1938180) | F: GCG TGG CAA CCG TGA AGG | | | |
|  |  |  | R: GCC TGG TGT CCA CAG ATC C | | | |
|  |  |  |  |  |  |  |
| Insulin-like binding-protein 4 | *igfbp4* | MN045298 | F: ACT CAG CGA TGG ACA GGC AGG AT | | | |
|  |  |  | R: CGG ATG TTG CTG TTG TTG GGA TGC T | | | |
|  |  |  |  |  |  |  |
| Insulin-like binding-protein 5b | *igfbp5b* | (LG15:3836279-3847001) | F: GTG CCA CTC CTT CCC AAA GAC AT | | | |
|  |  |  | R: CTG CTT GCC CAG CTT CCT CT | | | |
|  |  |  |  |  |  |  |
| Insulin-like binding-protein 6b | *igfbp6b* | (LG22-25:348158-350835) | F: CCA GGG ACC ATA ATG TTG CC | | | |
|  |  |  | R: TAC ACA CCA CAG GGC TCT C | | | |
|  |  |  |  | | | |
| Elongation of very long chain fatty acids 1 | *elovl1* | KF857295 | F: TAC ACA TCT TCC ACC ACT CCT TCA T | | | |
|  |  |  | R: CCA TTC CAC CAG GAG CAT AGG | | | |
|  |  |  |  |  |  |  |
| Elongation of very long chain fatty acids 4 | *elovl4* | KF857296 | F: ACC ATG CTT ACC GAC GCA AAC CTT | | | |
|  |  |  | R: CGA CGT GCT TGC CTC CCT TCT G | | | |
|  |  |  |  |  |  |  |
| Elongation of very long chain fatty acids 5 | *elovl5* | FR717358 | F: CAG TCA TGT ACC TTC TGA TCG TGT GGA TGG | | | |
|  |  |  | R: GGA GTA CGG CTG CCT GTG TTT CAT | | | |
| ^1^Obtained from GenBank database or the European seabass genome project (http://seabass.mpipz.mpg.de). Accession number of seabass genome is shown in parentheses. | | | | | | |
|  | | | | | | |
|  | | | | | | |
|  | | | | | | |
|  | | | | | | |
| **SUPPLEMENTARY TABLE 2 \|** (continued) | | | | | | |
| **Gene** | **Symbol** | **Accession number^1^** | **Primers** |  |  |  |
| Elongation of very long chain fatty acids 6 | *elovl6* | KF857297 | F: ACA TCA CCG TGC TGC TCT ACT CCT G | | | |
|  |  |  | R: CCG CCA CCT GGT CCT TGT AGC A | | | |
|  |  |  |  |  |  |  |
| Stearoyl-CoA desaturase 1b | *scd1b* | FN868643 | F: GCT TGT GGC ATA CTT CAT CCC TGG ACT C | | | |
|  |  |  | R: GGT GGC GTT GAG CAT CAC GGT GTA | | | |
|  |  |  |  |  |  |  |
| Fatty acid desaturase 2 | *fads2* | EU647692 | F: CCG CCG TGA CTG GGT GGA T | | | |
|  |  |  | R: GCA CAG GTA GCG AAG GTA GTA AGA CAT AGA | | | |
|  |  |  |  |  |  |  |
| Lipoprotein lipase | *lpl* | AM411614 | F: CAA TGT GAT CGT GGT GGA CTG | | | |
|  |  |  | R: CGT CGG GTA GTG CTG GTT | | | |
|  |  |  |  |  |  |  |
| Hepatic lipase | *hl* | KF857289 | F: CGC AGT GGC ACC AGC AAG A | | | |
|  |  |  | R: CGG CAT CCG AGA CCG TGT T | | | |
|  |  |  |  |  |  |  |
| Adipose triglyceride lipase | *atgl* | KF857294 | F: GGA GCC CTC ACT GCC ACT | | | |
|  |  |  | R: ATT CGC ACC AGT CTC TCC AAG A | | | |
|  |  |  |  |  |  |  |
| Hormone sensitive lipase | *hsl* | KF857293 | F: GCC CTG TCT CCA GAC TAT TGC TAT C | | | |
|  |  |  | R: GCT GCT ACA CCT ATT CCT GAC TGA T | | | |
|  |  |  |  | | | |
| Peroxisome proliferator-activated receptor α | *pparα* | AY590300 | F: CAG GAC ACG CAC AAC TCA ATC A | | | |
|  |  |  | R: GGA GAA CAC GGG ACA GTC AGA A | | | |
|  |  |  |  |  |  |  |
| Peroxisome proliferator-activated receptor γ | *pparγ* | AY590303 | F: CAG GAC ACG CAC AAC TCA ATC A | | | |
|  |  |  | R: GGA GAA CAC GGG ACA GTC AGA A | | | |
|  |  |  |  |  |  |  |
| Carnitine palmitoyltransferase 1a | *cpt1a* | KF857302 | F: TGC CAA GAG GTC ATC CAG AGT TCT | | | |
|  |  |  | R: AGT CCA CAT CAT CCG CCA GAG A | | | |
|  |  |  |  |  |  |  |
| Citrate synthase | *cs* | KF857304 | F: GTG TAT GAG ACC TCC GTG TTG G | | | |
|  |  |  | R: AGC AAC TTC TGA CAC TCT GGA ATG | | | |
|  |  |  |  |  |  |  |
| NADH dehydrogenase subunit 5 | *nd5* | KF857307 | F: CCC GAT TTC TGT GCC CTA CTA | | | |
|  |  |  | R: AGG AAA GGA GTG CCT GTG A | | | |
|  |  |  |  |  |  |  |
| Succinate dehydrogenase cytochrome b560 subunit | *sdhc* | KF857305 | F: ACA TGG GCA AGG GCT TCA AA | | | |
|  |  |  | R: CGA TGA TGG ACA GAC CGA TAA CG | | | |
|  |  |  |  |  |  |  |
| Cytochrome b | *cyb* | EF427553 | F: TGC CTA CGC TAT CCT TCG CTC GAT CC | | | |
|  |  |  | R: TAA CGC CAA CAC CCC GCC CAA T | | | |
| ^1^Obtained from GenBank database or the European seabass genome project (http://seabass.mpipz.mpg.de). Accession number of seabass genome is shown in parentheses. | | | | | | |
|  |  |  |  | | | |
|  |  |  |  | | | |
|  |  |  |  | | | |
| **SUPPLEMENTARY TABLE 2 \|** (continued) | | | | | | |
| **Gene** | **Symbol** | **Accession number^1^** | **Primers** | | | |
| Cytochrome c oxidase subunit I | *coxi* | KF857308 | F: ATA CTT CAC ATC CGC AAC CAT AA | | | |
|  |  |  | R: AAG CCT CCG ACT GTA AAT AAG AAA | | | |
|  |  |  |  |  |  |  |
| Cholesterol 7-alpha-monooxygenase | *cyp7a1* | KF857306 | F: TGC CAT CAA AGT CCC ACC TCT T | | | |
|  |  |  | R: CAC ATC ATA GGT AGG CTG GAG GAT TC | | | |
|  |  |  |  |  |  |  |
| Sirtuin 1 | *sirt1* | MH138004 | F: GGT GGA CCT CTT GAT TGT CAT TGG CTC TTC | | | |
|  |  |  | R: GGG ATG AGG GCA ACT GGT CGG ACT TTA | | | |
|  |  |  |  | | | |
| Sirtuin 2 | *sirt2* | MK983171 | F: TCT AAT TGA GGC TCA CGG AAC | | | |
|  |  |  | R: GAC GGG TAG ATT CTC TCC AAA G | | | |
|  |  |  |  |  |  |  |
| Mitochondrial respiratory uncoupling protein 1 | *ucp1* | MH138003 | F: CGA TTC CAA GCC CAG ACG AAC CT | | | |
|  |  |  | R: TGC CAG TGT AGC GAC GAG CC | | | |
|  |  |  |  |  |  |  |
| Mitochondrial respiratory uncoupling protein 3 | *ucp3* | (LG14:12134586-12136013) | F: CCA TGC TGA GAC AGG AAG GAC CCA CAT | | | |
|  |  |  | R: CCA GTC GCA GGA AAG AAG GCA TGA ACC | | | |
|  |  |  |  |  |  |  |
| Myoblast determination protein 1 | *myod1* | (LG6:934633-937237) | F: GAC CGA CCT GTC AGT CCA ACC G | | | |
|  |  |  | R: TGG AGT CTC GGA GAA ATA AGA GCT GTT GT | | | |
|  |  |  |  |  |  |  |
| Myoblast determination protein 2 | *myod2* | (LG5:26406310-26408511) | F: CTG CTG ATG ACC TCT ACG ATG AC | | | |
|  |  |  | R: GGC GTC CAG GTC GTC AAA | | | |
|  |  |  |  |  |  |  |
| Myogenic regulatory factor 4 | *mrf4* | (LGx:14305213-14306264) | F: GTC TCC TCT ATA CAA CGG CAA T | | | |
|  |  |  | R: CTG TCT CGG ACG GAA CAT TAT C | | | |
|  |  |  |  |  |  |  |
| Myogenic factor 5 | *myf5* | (LGx:14298644-14300040) | F: CGC AAC GCC ATC CAG TAC ATC G | | | |
|  |  |  | R: GCC GTA GTA GTT TTC CAC CTG CTC AT | | | |
|  |  |  |  |  |  |  |
| Myogenin | *myog* | (LG1A:13290583-13292182) | F: GAC CAA CCC TTA TTT CTT | | | |
|  |  |  | R: CAT CAT GGA GTT CCT ATC | | | |
| ^1^Obtained from GenBank database or the European seabass genome project (http://seabass.mpipz.mpg.de). Accession number of seabass genome is shown in parentheses. | | | | | | |
|  |  |  |  |  |  |  |
|  |  |  |  |  |  |  |
|  |  |  |  |  |  |  |
|  | | | | | | |
| **SUPPLEMENTARY TABLE 2 \|** (continued) | | | | | | |
| **Gene** | **Symbol** | **Accession number^1^** | **Primers** | | | |
| Myostatin | *mstn* | AY839106 | F: GCA GCA GCT TCT CGA CCA GTA | | | |
|  |  |  | R: ATC GTC GTC CTC CAT AAC CAC ATC | | | |
|  |  |  |  |  |  |  |
| Follistatin | *fst* | MK983166 | F: GTG CCA GTG ACA ACA CCA CAT ATC C | | | |
|  |  |  | R: ATC CCG AGT GCT TGA CTT CCA | | | |
|  |  |  |  |  |  |  |
| Fibroblast growth factor 4 | *fgf4* | (LG5:29962695-29967359) | F: GGC TTT GTG ACC GGA ATG G | | | |
|  |  |  | R: GTC CGC TGT CCC GTT CAG | | | |
|  |  |  |  |  |  |  |
| Fibroblast growth factor 6 | *fgf6* | AY831723 | F: CAA CGC CTA CGA GTC TCT GGT CTA C | | | |
|  |  |  | R: GCC ATG CTT GCT GAG TGC TAT GT | | | |
|  |  |  |  |  |  |  |
| Muscle RING-finger protein 1 | *murf1* | (UN:85299200-85300236) | F: TGG TGC GTC CTG TCA GTG | | | |
|  |  |  | R: CGG CTT GGT GAA CAT CTC AA | | | |
|  |  |  |  |  |  |  |
| Muscle atrophy F-box | *mafbx/atrogin-1* | MK983167 | F: ACT GAG GAC CGA CTG CTG TGG AAG A | | | |
|  |  |  | R: TGT CTG TCT GTG AAG TGG TAC TGG CAA AGT | | | |
|  |  |  |  |  |  |  |
| Myomaker | *mymk* | (LG20:21064893-21067975) | F: ATC TGT CTC TGG CTG TGT CCT TCA T | | | |
|  |  |  | R: CAG CAT TTC GTC CCG TCC CT | | | |
|  |  |  |  |  |  |  |
| Calpain 1 | *capn1* | FJ821591 | F: CTA CAG AGG AAA TCC GAC TAA GC | | | |
|  |  |  | R: CGG TCC ATT CCA CTT CCC | | | |
|  |  |  |  |  |  |  |
| Calpain 2 | *capn2* | MK983168 | F: AAC GAA CTG ACA TCC GAA CTG A | | | |
|  |  |  | R: ATT GCC GCT GTC ATC CAT CA | | | |
|  |  |  |  |  |  |  |
| Calpain 3 | *capn3* | MK983169 | F: ATA CCG ACG GGA CAG GGA AG | | | |
|  |  |  | R: GCT GCC ACG CCT TGA TCT T | | | |
|  |  |  |  |  |  |  |
| Calpastatin | *cpst* | MK983170 | F: AGA CGA CAC GCT GCC TCC A | | | |
|  |  |  | R: CTC AGT GGT TTA GGG ACA TCC TTG GGT TT | | | |
| ^1^Obtained from GenBank database or the European seabass genome project (http://seabass.mpipz.mpg.de). Accession number of seabass genome is shown in parentheses. | | | | | | |

| **SUPPLEMENTARY TABLE 3** \| Correlations between nutrients digestibility and balance, whole body composition, muscle FA composition and plasma metabolites with VSI, HSI, or *d*TM dose. | | | |
| --- | --- | --- | --- |
|  | **VSI** | **HSI** | ***d*TM dose** |
| ***Nutrients digestibility and balance*** |  |  |  |
| ADC of protein | NS | NS | -0.80 (< 0.01) |
| Digestible N intake | NS | NS | -0.78 (< 0.01) |
| N retention efficiency | NS | NS | 0.67 (0.02) |
| Fecal N losses | NS | NS | 0.93 (< 0.01) |
| Metabolic N losses | NS | NS | -0.84 (< 0.01) |
| Total N losses | NS | NS | -0.6 (< 0.01) |
| ADC of phosphorus | NS | NS | 0.92 (< 0.01) |
| P gain | NS | -0.59 (0.04) | NS |
| Digestible P intake | -0.62 (0.03) | NS | -0.84 (< 0.01) |
| Fecal P losses | NS | NS | -0.97 (< 0.01) |
| Total P losses | NS | NS | -0.6 (0.02) |
| L gain | 0.59 (0.04) | 0.59 (0.04) | 0.78 (< 0.01) |
| Lipids retention efficiency | 0.65 (0.02) | NS | NS |
| Fecal L losses | -0.64 (0.03) | NS | NS |
| Metabolic L losses | -0.64 (0.03) | -0.63 (0.03) | NS |
| Total L losses | -0.64 (0.03) | NS | NS |
| Digestible E intake | NS | NS | NS |
| E gain | NS | NS | 0.78 (< 0.01) |
| Branchial + urinary E losses | NS | NS | -0.84 (< 0.01) |
| ***Whole body composition*** |  |  |  |
| Moisture | NS | NS | -0.61 (0.04) |
| Lipids | NS | 0.59 (0.04) | 0.73 (< 0.01) |
| Energy | NS | NS | 0.73 (< 0.01) |
| Phosphorus | NS | -0.60 (0.04) | NS |
| ***Muscle FA composition*** |  |  |  |
| Σ MUFA | 0.78 (< 0.01) | 0.72 (< 0.01) | NS |
| n-3 LC-PUFA | NS | NS | -0.65 (0.02) |
| n-6 LC-PUFA | NS | 0.61 (< 0.01) | 0.97 (< 0.01) |
| ***Plasma metabolites*** |  |  |  |
| Triglycerides | 0.73 (< 0.01) | 0.85 (< 0.01) | 0.82 (< 0.01) |
| Cholesterol | 0.61 (0.04) | NS | NS |
| NEFA | NS | NS | 0.84 (< 0.01) |
| Values are *rs*-value (*p*-value of correlation). Differences were considered significant when *p* < 0.05. ADC, apparent digestibility coefficients; *d*TM, defatted *Tenebrio molitor* larvae meal; E, energy; FA, fatty acids; HSI, hepatosomatic index; L, lipids; LC-PUFA, long-chain polyunsaturated fatty acids; N, nitrogen; NEFA, non-esterified fatty acids; NS, not significant; P, phosphorus; VSI, viscerosomatic index. | | | |

| SUPPLEMENTARY TABLE 4 \| Intestinal morphological measurements of European seabass fed experimental diets | | | | | |
| --- | --- | --- | --- | --- | --- |
|  | CTRL | TM40 | TM80 | TM100 | *p-*value |
| Cross-sectional area, mm^2^ | 14.5 ± 1.6 | 11.1 ± 0.7 | 12.6 ± 0.9 | 12.7 ± 0.6 | 0.34 |
| *Muscularis* externa, µm | 97.9 ± 4.3 | 110.6 ± 3.7 | 96.4 ± 1.3 | 89.4 ± 2.5 | 0.61 |
| Outer longitudinal layer, µm | 34.3 ± 8.8 | 41.4 ± 9.3 | 35.2 ± 9.4 | 31.6 ± 3.6 | 0.12 |
| Inner circular layer, µm | 63.6 ± 2.9 | 69.3 ± 3.1 | 61.2 ± 3.1 | 57.8 ± 1.2 | 0.12 |
| Submucosa, μm | 26.5 ± 0.7^c^ | 29.2 ± 1.0^b^ | 30.2 ± 1.0^ab^ | 32.8 ± 1.0^a^ | < 0.01 |
| Lamina propria, μm | 27.3 ± 1.4 | 28.4 ± 1.6 | 29.3 ± 0.4 | 30.3 ± 2.3 | 0.62 |
| *Villus* length, µm | 2000.6 ± 83.6 | 1749.1 ± 68.8 | 1833.6 ± 83.2 | 1821.0 ± 53.5 | 0.12 |
| *Villus* width, µm | 179.9 ± 11.4 | 160.5 ± 9.5 | 176.3 ± 11.0 | 162.6 ± 5.6 | 0.44 |
| Goblet cells, nº per *villus* | 304.3 ± 36.0 | 227.8 ± 20.8 | 284.3 ± 19.0 | 228.7 ± 22.4 | 0.21 |
| Acid goblet cells, nº per *villus* | 191.8 ± 25.8 | 157.4 ± 6.6 | 178.4 ± 9.6 | 147.1 ± 10.3 | 0.07 |
| Neutral goblet cells, nº per *villus* | 112.5 ± 12.5 | 70.4 ± 9.8 | 105.9 ± 12.3 | 81.6 ± 1.6 | 0.09 |
| Values are means ± SEM; n = 9 (3 fish/replicate). Labeled means without a common superscript letter differ significantly, *p* < 0.05. | | | | |  |

| **SUPPLEMENTARY TABLE 5 \|** Muscular cellularity parameters of European seabass fed experimental diets | | | | |  |
| --- | --- | --- | --- | --- | --- |
|  | CTRL | TM40 | TM80 | TM100 | *p-*value |
| White muscle cross-sectional area, mm^2^ | 489.7 ± 12.8 | 495.7 ± 12.43 | 458.5 ± 13.8 | 458.74 ± 19.27 | 0.17 |
| Total n° of fibers × 10^3^ | 107.4 ± 42.0 | 107.5 ± 50.8 | 104.0 ± 54.0 | 105.0 ± 68.1 | 0.96 |
| Fibers density (n°/mm^2^) | 219.6 ± 7.86 | 216.8 ± 8.3 | 227.2 ± 10.6 | 228.1 ± 9.8 | 0.78 |
| Fibers diameter, μm | 66.7 ± 3.9 | 66.7 ± 1.1 | 65.5 ± 1.10 | 65.41 ± 1.1 | 0.82 |
| Fibers ≤ 20 μm, % | 6.3 ± 0.01 | 5.0 ± 0.004 | 5.4 ± 0.01 | 6.25 ± 0.004 | 0.39 |
| Fibers > 140 μm, % | 2.2 ± 0.01 | 3.2 ± 0.01 | 2.2 ± 0.004 | 2.64 ± 0.01 | 0.54 |
| Values are means ± SEM; n = 9 (3 fish/replicate). CSA, cross-sectional area. | | | | |  |

| SUPPLEMENTARY TABLE 6 \| Muscle texture profile and color of European seabass fed experimental diets | | | | | |
| --- | --- | --- | --- | --- | --- |
|  | CTRL | TM40 | TM80 | TM100 | *p-*value |
| *Texture profile* | | | | | |
| Hardness, N | 0.9 ± 0.04 | 1.0 ± 0.06 | 1.0 ± 0.05 | 0.9 ± 0.05 | 0.34 |
| Adhesiveness, J | -0.01 ± 0.002 | -0.004 ± 0.001 | -0.004 ± 0.002 | -0.005 ± 0.001 | 0.40 |
| Springiness | 1.2 ± 0.1 | 1.4 ± 0.1 | 1.6 ± 0.1 | 1.3 ± 0.1 | 0.29 |
| Cohesiveness | 0.4 ± 0.01 | 0.5 ± 0.02 | 0.5 ± 0.02 | 0.5 ± 0.02 | 0.83 |
| Chewiness, J | 0.5 ± 0.1 | 0.7 ± 0.1 | 0.8 ± 0.1 | 0.6 ± 0.1 | 0.16 |
| Resilience | 0.4 ± 0.1 | 0.4 ± 0.1 | 0.4 ± 0.1 | 0.4 ± 0.1 | 0.55 |
| *Color* |  |  |  |  |  |
| L* | 40.7 ± 0.5 | 40.8 ± 0.4 | 40.8 ± 0.5 | 41.7 ± 0.5 | 0.41 |
| a* | -0.2 ± 0.1 | -0.5 ± 0.1 | -0.3 ± 0.1 | -0.3 ± 0.1 | 0.21 |
| b* | -0.7 ± 0.3 | -1.0 ± 0.2 | -1.0 ± 0.1 | 0.1 ± 0.4 | 0.25 |
| C*^1^ | 1.2 ± 0.1 | 1.2 ± 0.1 | 1.1 ± 0.1 | 1.0 ± 0.2 | 0.81 |
| H°^2^ | 222.7 ± 22.5 | 238.8 ± 8.1 | 250.2 ± 4.2 | 182.8 ± 22.9 | 0.40 |
| Values are means ± SEM; n = 9 (3 fish/replicate). a*, redness; b*, yellowness; C*, chroma; H°, hue; L*, lightness. Chroma = (a* 2 + b* 2)1/2; Hue = tan−1 (b* / a*). | | | | | |

| **SUPPLEMENTARY TABLE 7 \|** Relative expression of hepatic genes of European seabass fed experimental diets | | | | | |
| --- | --- | --- | --- | --- | --- |
|  | CTRL | TM40 | TM80 | TM100 | *p-*value |
| *ghr-i* | 6.6 ± 0.3 | 7.0 ± 0.7 | 6.2 ± 0.6 | 7.5 ± 0.5 | 0.43 |
| *ghr-ii* | 2.3 ± 0.3 | 2.5 ± 0.1 | 2.2 ± 0.3 | 2.1 ± 0.4 | 0.77 |
| *igf-i* | 20.7 ± 1.3 | 21.9 ± 1.9 | 18.7 ± 1.6 | 18.5 ± 1.7 | 0.40 |
| *igf-ii* | 4.1 ± 0.6 | 4.4 ± 0.5 | 3.3 ± 0.6 | 3.5 ± 0.9 | 0.66 |
| *igfbp1b* | 1.5 ± 0.3 | 1.8 ± 0.3 | 1.2 ± 0.3 | 1.5 ± 0.5 | 0.70 |
| *igfbp2b* | 26.3 ± 2.3 | 30.0 ± 3.4 | 28.2 ± 2.6 | 28.1 ± 3.1 | 0.80 |
| *igfbp4* | 4.1 ± 0.5 | 4.1 ± 0.2 | 4.3 ± 0.4 | 3.8 ± 0.6 | 0.30 |
| *elovl1* | 0.31 ± 0.1 | 0.3 ± 0.04 | 0.3 ± 0.02 | 0.4 ± 0.04 | 0.64 |
| *elovl4* | 0.01 ± 0.0001 | 0.001 ± 0.0002 | 0.0001 ± 0.0002 | 0.0001 ± 0.0001 | 0.60 |
| *elovl5* | 0.01 ± 0.0001 | 0.01 ± 0.00001 | 0.01 ± 0.00001 | 0.01 ± 0.00001 | 0.20 |
| *elovl6* | 6.2 ± 1.2^a^ | 4.3 ± 0.8^ab^ | 4.1 ± 1.02^ab^ | 2.4 ± 0.4^b^ | 0.02 |
| *scd1b* | 1.5 ± 0.5 | 0.7 ± 0.1 | 0.7 ± 0.2 | 0.8 ± 0.1 | 0.08 |
| *fads2* | 0.3 ± 0.1^b^ | 0.2 ± 0.1^b^ | 0.3 ± 0.08^ab^ | 1.0 ± 0.3^a^ | 0.02 |
| *lpl* | 4.3 ± 0.5 | 4.8 ± 0.4 | 3.9 ± 0.3 | 4.4 ± 0.6 | 0.57 |
| *hl* | 4.8 ± 0.3 | 5.4 ± 0.4 | 5.8 ± 0.4 | 6.2 ± 0.8 | 0.43 |
| *atgl* | 1.4 ± 0.2 | 1.6 ± 0.2 | 1.8 ± 0.2 | 1.7 ± 0.2 | 0.41 |
| *hsl* | 1.0 ± 0.1 | 1.1 ± 0.1 | 0.9 ± 0.04 | 1.0 ± 0.1 | 0.29 |
| *pparα* | 0.34 ± 0.1 | 0.33 ± 0.03 | 0.32 ± 0.1 | 0.2 ± 0.03 | 0.09 |
| *pparγ* | 0.4 ± 0.03 | 0.5 ± 0.1 | 0.4 ± 0.03 | 0.5 ± 0.1 | 0.26 |
| *cpt1a* | 0.8 ± 0.1 | 0.8 ± 0.04 | 0.7 ± 0.05 | 0.8 ± 0.1 | 0.91 |
| *cs* | 1.9 ± 0.1 | 2.0 ± 0.1 | 1.8 ± 0.2 | 1.8 ± 0.2 | 0.60 |
| *nd5* | 86.8 ± 6.2 | 111.1 ± 6.1 | 98.7 ± 7.7 | 105.4 ± 10.6 | 0.18 |
| *sdhc* | 2.8 ± 0.2 | 3.0 ± 0.1 | 2.9 ± 0.1 | 3.0 ± 0.3 | 0.84 |
| *cyb* | 0.2 ± 0.01 | 0.2 ± 0.01 | 0.2 ± 0.03 | 0.2 ± 0.01 | 0.27 |
| *coxi* | 279.9 ± 31.5 | 324.9 ± 19.3 | 283.0 ± 25.1 | 355.9 ± 46.9 | 0.41 |
| *cyp7a1* | 6.9 ± 0.7^a^ | 4.2 ± 0.7^b^ | 3.0 ± 0.5^b^ | 3.2 ± 0.5^b^ | <0.01 |
| *sirt1* | 0.1 ± 0.01 | 0.1 ± 0.01 | 0.2 ± 0.01 | 0.2 ± 0.01 | 0.16 |
| *sirt2* | 0.8 ± 0.04 | 0.9 ± 0.04 | 0.8 ± 0.1 | 0.9 ± 0.1 | 0.22 |
| *ucp1* | 7.2 ± 0.6 | 8.2 ± 1.6 | 5.8 ± 0.6 | 5.8 ± 0.6 | 0.49 |
| Values are means ± SEM; n = 9 (3 fish/replicate). Labeled means without a common superscript letter differ significantly. | | | | | |

| **SUPPLEMENTARY TABLE 8 \|** Relative expression of muscle genes of European seabass fed experimental diets | | | | | |
| --- | --- | --- | --- | --- | --- |
|  | CTRL | TM40 | TM80 | TM100 | *p-*value |
| *ghr-i* | 1.54 ± 0.22 | 1.41 ± 0.09 | 1.28 ± 0.07 | 1.47 ± 0.11 | 0.63 |
| *ghr-ii* | 1.10 ± 0.18 | 1.29 ± 0.16 | 1.21 ± 0.15 | 1.29 ± 0.20 | 0.85 |
| *igf-i* | 0.04 ± 0.01 | 0.04 ± 0.01 | 0.03 ± 0.01 | 0.04 ± 0.01 | 0.72 |
| *igf-ii* | 0.87 ± 0.11 | 0.72 ± 0.05 | 0.69 ± 0.04 | 0.69 ± 0.04 | 0.24 |
| *igfbp3a* | 1.74 ± 0.14 | 1.82 ± 0.22 | 1.84 ± 0.12 | 1.72 ± 0.15 | 0.95 |
| *igfbp5b* | 2.27 ± 0.29 | 2.65 ± 0.27 | 2.88 ± 0.44 | 2.84 ± 0.20 | 0.47 |
| *igfbp6b* | 0.29 ± 0.02 | 0.30 ± 0.03 | 0.32 ± 0.02 | 0.33 ± 0.02 | 0.56 |
| *scd1b* | 0.72 ± 0.08 | 0.63 ± 0.08 | 0.70 ± 0.08 | 0.67 ± 0.07 | 0.84 |
| *cpt1a* | 2.83 ± 0.33 | 2.75 ± 0.27 | 2.83 ± 0.26 | 2.91 ± 0.34 | 0.98 |
| *cs* | 23.29 ± 2.35 | 23.78 ± 1.21 | 21.76 ± 1.40 | 24.83 ± 1.59 | 0.64 |
| *sirt1* | 0.28 ± 0.02 | 0.28 ± 0.03 | 0.27 ± 0.02 | 0.30 ± 0.02 | 0.87 |
| *sirt2* | 1.41 ± 0.11 | 1.35 ± 0.12 | 1.41 ± 0.16 | 1.32 ± 0.05 | 0.92 |
| *ucp3* | 3.01 ± 0.29 | 3.12 ± 0.30 | 2.79 ± 0.64 | 2.90 ± 0.34 | 0.35 |
| *myod1* | 8.43 ± 0.88 | 8.78 ± 0.64 | 8.63 ± 0.82 | 7.43 ± 0.55 | 0.57 |
| *myod2* | 3.32 ± 0.23^a^ | 3.23 ± 0.17^a^ | 2.63 ± 0.31^b^ | 2.65 ± 0.11^b^ | 0.03 |
| *mrf4* | 0.45 ± 0.06 | 0.48 ± 0.07 | 0.40 ± 0.04 | 0.41 ± 0.03 | 0.70 |
| *myf5* | 0.33 ± 0.02 | 0.32 ± 0.02 | 0.30 ± 0.02 | 0.32 ± 0.01 | 0.63 |
| *myog* | 2.42 ± 0.24 | 2.34 ± 0.19 | 2.30 ± 0.14 | 2.46 ± 0.15 | 0.91 |
| *mstn* | 0.45 ± 0.09^a^ | 0.61 ± 0.15^ab^ | 0.81 ± 0.16^ab^ | 1.04 ± 0.23^b^ | 0.01 |
| *fst* | 0.20 ± 0.03 | 0.19 ± 0.02 | 0.19 ± 0.02 | 0.20 ± 0.02 | 0.98 |
| *fgf4* | 0.28 ± 0.02 | 0.30 ± 0.03 | 0.30 ± 0.01 | 0.31 ± 0.02 | 0.83 |
| *fgf6* | 0.86 ± 0.06 | 0.73 ± 0.08 | 0.85 ± 0.09 | 0.81 ± 0.04 | 0.53 |
| *murf1* | 15.23 ± 1.51 | 15.84 ± 1.89 | 15.56 ± 1.86 | 15.71 ± 0.61 | 0.99 |
| *mafbx/atrogin 1* | 1.73 ± 0.27^a^ | 1.38 ± 0.48^ab^ | 1.47 ± 0.49^ab^ | 0.92 ± 0.14^b^ | 0.01 |
| *mymk* | 0.07 ± 0.01^b^ | 0.08 ± 0.01^ab^ | 0.09 ± 0.02^ab^ | 0.10 ± 0.01^a^ | 0.04 |
| *capn1* | 1.20 ± 0.09 | 1.04 ± 0.07 | 1.12 ± 0.09 | 1.22 ± 0.06 | 0.36 |
| *capn2* | 1.02 ± 0.09 | 1.08 ± 0.10 | 1.08 ± 0.09 | 1.10 ± 0.06 | 0.92 |
| *capn3* | 5.33 ± 0.41 | 5.35 ± 0.29 | 5.93 ± 0.68 | 5.43 ± 0.43 | 0.96 |
| *cpst* | 0.87 ± 0.04 | 0.87 ± 0.07 | 0.90 ± 0.08 | 0.95 ± 0.04 | 0.72 |
| Values are means ± SEM; n = 9 (3 fish/replicate). Labeled means without a common superscript letter differ significantly. | | | | | |

| **SUPPLEMENTARY TABLE 9 \|** Correlations between expression of hepatic genes and plasma metabolites | | | | | |
| --- | --- | --- | --- | --- | --- |
|  | Glucose | Protein | Triglycerides | Cholesterol | NEFA |
| *elovl6* | NS | -0.41 (0.04) | NS | -0.64 (< 0.01) | NS |
| *fads2* | NS | NS | NS | 0.38 (0.04) | NS |
| *scd1b* | NS | NS | 0.49 (0.04) | NS | NS |
| *cyp7a1* | 0.37 (0.02) | NS | NS | -0.46 (< 0.01) | NS |
| *pparα* | NS | NS | NS | -0.37 (0.02) | NS |
| Values are *rs*-value (*p*-value of correlation); Differences were considered significant when *p* < 0.05. NEFA, non-esterified fatty acids; NS, not significant. | | | | | |
